# Proceedings of the 6th National Patient Reported Outcome Measures (PROMs) Annual UK Research Virtual Conference, Bridgend, Wales 2022: 14–15 June 2022, Virtual, Bridgend, Wales, UK

**DOI:** 10.1007/s11136-023-03353-w

**Published:** 2023-03-20

## Introduction

### Proceedings of the 6th National Patient Reported Outcome Measures (PROMs) Annual UK Research Virtual Conference, Bridgend, Wales 2022

#### Kathleen Withers^1,2^, Sarah Puntoni^2^,Lawrence Humphreys^2^, Samantha Harris^2^, Gareth Rosser^2^, Timothy Pickles ^3^, Amanda Lane ^4^, Patricia Holch ^5^, Jon Evans^6^, Grace Turner ^7,8,9^

#### ^1^Cedar Health Technology Research Centre, Cardiff and Vale UHB, United Kingdom. ^2^ Welsh Value in Health Centre, Cwm Taf Morgannwg UHB, United Kingdom.^3^ Centre for Trials Research, College of Biomedical and Life Sciences, Cardiff University, Cardiff, United Kingdom. ^4^ School of Health and Related Research, University of Sheffield, Sheffield, United Kingdom. ^5^ Psychology Department, Leeds School of Social Sciences, Leeds Beckett University, Leeds, United Kingdom. ^6^ Health and Policy Research Group, University of Exeter, Exeter, United Kingdom. ^7^ Institute of Applied Health Research, University of Birmingham, Birmingham, United Kingdom. ^8^ Centre for Patient Reported Outcomes Research, University of Birmingham, Birmingham, United Kingdom. ^9^ NIHR Surgical Reconstruction and Microbiology Research Centre, University Hospitals Birmingham NHS Foundation Trust and University of Birmingham, Birmingham, United Kingdom.

The 6th UK Patient Reported Outcome Measures (PROMs) Research Conference was held on the 14^th^ and 15^th^ of June 2022. Hosted online, it attracted an international audience from locations including Europe, the USA, Australia, New Zealand, and the Caribbean, and was attended by 381 people with representatives from academia, healthcare, patients, and industry. Previously hosted by the Universities of Sheffield, Oxford, Birmingham and Leeds Beckett, for the first time it was hosted by a non-academic organisation, making its inaugural visit to Wales, and was hosted by The Welsh Value in Health Centre. Welcoming abstracts on any topics, focused themes included: Methods; Implementation; Palliative Care; COVID-19; Patient and Public Involvement; Mental Health; and Social Care.

## Conference Summary

Due to the ongoing uncertainty related to COVID-19, this event was held online as it had been in 2021. The conference included plenary sessions, expert panels and oral presentations from PROMs researchers across the UK and beyond. As well as expert speakers, the abstract submissions led to 24 oral presentations and 35 posters presentations.

Highlights from the two days included: **Professor Hamish Laing** from the Value Based Health and Care Academy at Swansea University gave a presentation exploring the ways that PROMs are being deployed in episodic and long-term care, and across the data collection time course. This included their use as a symptom tracking mechanism, as a population needs assessment, and as a component in outcome-based procurement. **Dr Ellen Elsman** gave a presentation on how to select an outcome measurement instrument, referencing the COSMIN initiative. This was followed by an expert panel with **Alice Andrews**, **Professor Hamish Laing**, **Dr Sabina De Rosis**, and **Allan Wardaugh**, discussing how to embed PROMs in direct care from a system perspective, with a focus on system interoperability, the digital landscape and date visualisation. A second expert panel discussed embedding PROMs in direct care from the clinical perspective. Involving **Linda Edmunds**, **Dr Peter Hall**, **Sioned Jones**, **Dr Mohid S Khan**, **Anji Kingman**, **Mr D. Phill Thomas**, **Dr Sally Lewis**, and **Katie Spencer**, this session looked at the application of PROMs in different condition, and the panel shared lessons learnt from their own experiences. **Professor Alf Collins**, NHS England’s Clinical Director, examined how PROMs can help unlock person centred care and facilitate the shift towards support patients’ in discussing ‘what matters to you?’.

The conference offered five prizes which were judged by a Scientific Committee with representatives from Academic NHS Wales and Third Sector organisations/ Patient representatives, and awarded as follow:

**Most Promising Early or PhD Abstract Submission:** James Glasbey, NIHR Global Health Research Unit on Global Surgery at the University of Birmingham, UK.

**Patient and Public Involvement Award:** Dr Ameeta Retzer, University of Birmingham, UK.

**Value-based Principles Award:** Dr Geraint Palmer from Cardiff University, and Dr Robert Palmer from Cedar Health Technology Research Centre, Cardiff and Vale UHB, UK.

**Equality, Diversity and Inclusivity Award:** Dr Ameeta Retzer, University of Birmingham, UK.

**Best Overall Award:** James Glasbey, NIHR Global Health Research Unit on Global Surgery at the University of Birmingham, UK.

**Ethics declarations**

Conflict of interest:

This conference was funded by the Welsh Value in Health Centre. Oxford University Innovations Ltd. funded the conference award prizes.

Consent for publication: Informed consent was obtained.

## Abstracts for oral presentation

## Abstract Session 1A

## A1 published 10.1186/s13063-021-05398-z

## A2 Long COVID Rehabilitation Services, Cardiff and Vale and Cwm Taf Morgannwg University health Boards: Social Return on Investment

### *^1^Megan Dale, ^1^Rachel Wallbank, ^2^Emma Ralph, ^1^Robert Letchford, ^2^Sofia Harries, ^1^Aura Frizzati, ^1^Robert Palmer, ^1^Kathleen Withers

#### ^1^Cardiff and Vale University Health Board, Cardiff, United Kingdom. ^2^Cwm Taf Morgannwg University Health Board, Mountain Ash, United Kingdom

Cardiff and Vale and Cwm Taf Morgannwg University Health Boards introduced Long COVID Rehabilitation Services to support people with Long COVID between December 2020 and January 2021 as a response to the Covid-19 pandemic and the emergence of patients with symptoms following COVID-19 infection. The multi-disciplinary teams include physiotherapists, occupational therapists, speech and language therapists, dieticians, and for some teams, psychologists and GPs. The services provide an initial one-to-one assessment which may be followed by further individual interventions, and in some cases group interventions. These aim to provide self-management advice and techniques for rehabilitation. The services were initially funded by individual health boards, but subsequently received additional funding from the Welsh Government as part of the ‘Adferiad’ (Recovery) programme. Cedar Health Technology Research Centre carried out a Social Return on Investment (SROI) for both of these health boards, linked to Cedar’s work at national level together with Welsh Value in Health Centre (WViHC) and all Local Health Boards. Social Return on Investment (SROI) is a method of evaluating the impact of a service, by measuring changes that are relevant to the people or organisations that experience or contribute to the service. This is used to understand where the value lies, who experiences that value and its importance to them, as well as calculating a ratio of benefits to costs. Cedar used the nationally collected patient-reported outcome and experience measures (PROMs and PREMs), together with interviews, SROI specific surveys and group discussions to gather views from stakeholders, primarily service users, but also their families, service providers, GPs and an employer. The key outcomes reported by service users were the feeling of being listened to, understood and believed, and meeting (virtually) other people who were going through similar experiences. “somebody listening and understanding, believing in you. Being referred to the hub gave my condition legitimacy—taken seriously.“ “all of a sudden it’s like—I'm not making this up, there are actually other people who feel the same as me.” For some people the impact of these was a turning point in how they felt they were coping. People mentioned learning to pace themselves and not try to “push through”, and how information such as an occupational therapist’s plan for return to work could help them cope. “me telling them I need a really slow phase to return isn’t the same as somebody in a healthcare position telling them.”

## A3 The Clinical Meaning of Family Reported Outcome Measure (FROM-16) Scores: Translational Research to Support Holistic Clinical Practice

### *^1^Rubina Shah, ^2^Faraz M Ali, ^2^Stuart J Nixon, ^3^Kennedy Otwombe, ^1^John R Ingram, ^4,5^Sam S Salek, ^1^Andrew Y Finlay

#### ^1^Cardiff University, Cardiff, United Kingdom. ^2^MS society Cardiff, Cardiff, United Kingdom. ^3^University of the Witwatersrand, Johannesburg, South Africa. ^4^University of Hertfordshire, Hatfield, United Kingdom. ^5^Institute of Medicines Development, Cardiff, United Kingdom

Background: The major secondary burden of having a partner or family member with a health condition is often ignored, but now can be measured with the generic Family Reported Outcome Measure (FROM-16). Family burden data may contribute to assessment in value-based healthcare research. Aim: The aim was to develop score bands using the anchor-based approach in order to assign clinical meaning to FROM-16 scores. Methods: A cross-sectional online study recruited family members of patients with different health conditions through 58 UK-based patient support groups, research support platforms (HealthWise Wales, Autism Research Centre Cambridge University database, Join Dementia Research) and Welsh social services departments. Family members completed the FROM-16 and a 5-point Likert scale Global Question (GQ) concerning overall impact of their relative's health condition on their quality of life. Multiple FROM-16 band sets were devised by mapping mean, median and mode of the GQ scores against each FROM-16 score and ROC-AUC cut off values. The band set with the best agreement with GQ score based on weighted Kappa (WK) was selected. Results: A total of 4,413 family members/partners (male = 1533, 34.7%; female = 2858, 64.8%, unknown = 16, 0.4%; other = 6, 0.14%) of patients (male = 1994, 45.2%; female = 2400, 54.4%; unknown = 12, 0.3%; other = 7, 0.16%) with > 200 health conditions across 27 medical specialities completed the survey: mean FROM-16 score = 15.02 (range 0–32, SD = 8.08), mean GQ score = 2.32 (range 0–4, SD = 1.08). The proposed FROM-16 score bands are 0–1 = no effect on family member; 2–8 = small effect; 9–16 = moderate effect; 17–25 = very large effect; 26–32 = extremely large effect on family members (WK = 0.596). Conclusions: The resultant FROM-16 score bands provide new information to clinicians and researchers about how to clinically interpret scores and score changes, allowing better informed treatment decisions for patients and their families. The FROM-16’s score bands and short administration time demonstrate its potential to support clinical practice and health service research. The now meaningful information from the use of FROM-16 can be used to measure and understand more globally the wider burden of disease.

## A4 Patient-centred outcome measure design: the perspectives and preferences of children and young people with life-limiting or life-threatening conditions

### *^1^Daney Harðardóttir, ^1,2^Lucy Coombes, ^1^Debbie Braybrook, ^1^Hannah Scott, ^1^Anna Roach, ^1^Katherine Bristowe, ^1^Clare Ellis-Smith, ^3^Julia Downing, ^4,5^Myra Bluebond-Langner, ^6^Joanna Laddie, ^7^Michelle Hills, ^8^Christina Ramsenthaler, ^9^Lorna K Fraser, ^10^Fliss EM Murtagh, ^1^Richard Harding

#### ^1^Florence Nightingale Faculty of Nursing Midwifery and Palliative Care, King’s College London, Cicely Saunders Institute of Palliative Care, Policy and Rehabilitation, London, United Kingdom. ^2^Royal Marsden NHS Foundation Trust, London, United Kingdom. ^3^International Children’s Palliative Care Network, Kampala, Uganda. ^4^University College London, Louis Dundas Centre for Children’s Palliative Care, London, United Kingdom. ^5^Rutgers University, New Jersey, USA. ^6^Evelina London Children's Hospital, Guy's & St Thomas’ NHS Foundation Trust, London, United Kingdom. ^7^Martin House Children's Hospice, Wetherby, United Kingdom. ^8^Hull York Medical School, University of Hull, Hull, United Kingdom. ^9^Martin House Research Centre, Department of Health Sciences, University of York, York, United Kingdom. ^10^Wolfson Palliative Care Research Centre, Hull York Medical School, University of Hull, Hull, United Kingdom

Background: Children and young people (CYP) with life-limiting or life-threatening conditions (LLLTC) face specific challenges when self-reporting health outcomes, including communication difficulties and sensitivities around subject matter. No ideal self-reported patient-centred outcome measure (PCOM) currently exists for this population. Practical aspects of design need to be considered in line with CYP’s preferences and capabilities to ensure meaningful participation in measurement, and to enable child- and family-centred care. Aims: To identify preferences for PCOM response format, recall period, administration mode, and length, among CYP with LLLTC. Methods: Semi-structured qualitative interviews with CYP aged 5–17 years with LLLTC. CYP were purposively sampled from nine UK sites. Verbatim transcripts were analysed in NVivo using Framework analysis with inductive and deductive coding. Results: 26 CYP with a range of LLLTC (primary diagnosis: 10 gastrointestinal, 6 cancer, 5 neurological, 3 congenital, 1 metabolic, 1 respiratory) were interviewed. Response format: many participants reported familiarity with numeric response scales, especially for pain. However, most preferred response formats with pictures, most often emojis. Children under 10 years old in particular preferred emojis, while preferences among older CYP were more variable. Recall period: Participants preferred a short recall, either because they cannot remember far back, or they do not want to think about past ill health. Most felt that they could report health-related outcomes from between the past day up to the past week. Older CYP tended to favour longer recall periods compared to younger children. Administration mode: whilst most participants preferred to complete measures electronically or had no preference, a small number had a strong preference for paper-based measures, suggesting PCOMs should be available in multiple formats. Length: ten or fewer questions were preferred. Conclusions: CYP with LLLTC interviewed are accustomed to answering questions about their own health and can communicate preferences to inform PCOM design. Generally, they prefer visually appealing response formats, short measures, and electronic administration. Importantly, respondent burden needs to be considered at the design stage, as demonstrated by preferences for a brief measure and short recall period. The results presented have practical implications for design and development of PCOMs for CYP with LLLTC, whose voices must be included early in measure development to ensure acceptability, feasibility, and enhance valid and reliable self-report. Funding: European Research Council [Grant ID: 772635].

## Abstract Session 1B

## A5 published 10.1016/j.jval.2021.11.1136

## A6 Feasibility and implementation of the MYCaW® person centred outcome measure within a NHS frailty service

### *^1^Helen Seers, ^2^Joanne Appleton, ^2^Sally-Anne Bauer, ^2^Christine Cam, ^2^Gail Pasquall, ^1^Marie Polley

#### ^1^Meaningful Measures Ltd, Bristol, United Kingdom. ^2^NHS England and NHS Improvement – South West, Bristol, United Kingdom

Introduction: This project aimed to investigate the feasibility of using the MYCaW® tool (Measure Yourself Concerns and Wellbeing) within a NHS frailty service, to provide greater insight into the specific needs of people living with mild, moderate and severe frailty (as defined by the Rockwood Clinical Scale—RCFS). MYCaW® is a short tool, which is routinely incorporated into consultations to understand and prioritise needs and concerns. The tool enables the individual to assign a score to the problem/concern and their wellbeing. Follow-up enables measurement of changes in reported concerns and wellbeing over time. Method: Participants were recruited either through use of Complex Care at Home Service (CC@H) provided by Gloucestershire Health and Care Foundation Trust, or South Cotswolds Frailty Service (SCFS), an anticipatory care community service delivered by the South Cotswolds Primary Care Network (PCN). RCFS and MYCaW® data were collected by service staff. Data was also collected from healthcare practitioners about their experience of using MYCaW®. Results: 310 people (257 from CC@H and 53 from SCFS) completed the baseline questionnaire and 113 people provided follow-up MYCaW® concern and wellbeing scores data. The modal person was 85–89 years old and female. Despite experiencing severe frailty, patients’ designated MYCaW® concerns scores showed statistically significant improvements, and a high percentage of people (71%) had clinically significant levels of score changes. There was a statistically significant mean improvement in wellbeing scores. When that data was stratified and analysed according to RCFS severity, concerns improved regardless of level of severity of frailty, but wellbeing only statistically improved for people experiencing mild frailty. RCFS scores did not change over time. Conclusion: A bespoke MYCaW® frailty coding framework was created by revising the existing coding framework for MYCaW®. This framework provides a standardised yet rich picture of the concerns that are important to patients experiencing frailty. The five most frequent concerns related to mobility, managing the household and activities of daily living (ADLs), physical problems, housing and independence. The top concerns for mild and moderate frailty were physical (mobility) and the top concern for severe frailty was ADL. Importantly, this information can be used by healthcare practitioners to improve the personalised nature of the support they provide. The MYCaW® tool was implemented in the services’ systems and data was successfully collected from a fragile cohort during the Covid-19 pandemic. Staff experience of using MYCaW® showed that the measure was acceptable and worked well in practice.

## A7 Co-creation of a Patient Reported Outcome Measure for Older People with frailty and Acute Care needs (PROM-OPAC)

### *^1,2^James van Oppen, ^3^Simon Conroy, ^4^Jose M Valderas, ^1,2^Timothy Coats, ^1^Nicola Mackintosh

#### ^1^University of Leicester, Leicester, United Kingdom. ^2^University Hospitals of Leicester NHS Trust, Leicester, United Kingdom. ^3^University College London, London, United Kingdom. ^4^National University Health System, Singapore, Singapore

Background: Older people living with frailty have unique outcome goals for acute healthcare, classified in previous qualitative work as ‘Autonomy’ and ‘Function’. A systematic review has identified Patient-Reported Outcome Measures (PROMs) which adequately measure ‘Function’ during acute illness. Existing acute care PROMs do not comprehensively consider ‘Autonomy’. Historically, research has often excluded older people living with frailty, both as partners and as participants. This study used a co-creation process to draft and evaluate items for a PROM for Older People living with frailty and Acute Care needs (PROM-OPAC). Co-creation grounded ideation, design, and evaluation in lay perspectives. Methods: The study was steered by three lay research partners who were older and living with or caring for people with frailty. Potential novel ‘Autonomy’ items were drafted with the lay partners. The novel ‘Autonomy’ items and existing ‘Function’ measures were appraised for face validity by lay members of an ageing-specialised Patient and Public Involvement forum. Retained items were then evaluated for content validity in cognitive interviews with patient participants who were older, living with frailty, and receiving acute healthcare. Finally, reduced and improved ‘Autonomy’ items were integrated with the best available ‘Function’ measure to form a comprehensive draft instrument. Results: Twenty-eight novel ‘Autonomy’ items were drafted, and four existing ‘Function’ measures were appraised. Assessment of face validity was by four lay PPI forum members and of content validity was with fourteen patient participants. Seven ‘Autonomy’ items were retained, and for ‘Function’ the EQ-5D-5L had the best balance of content validity and accessibility. Conclusion: Older people who were living with frailty contributed substantially to the co-creation of a new PROM. They were engaged as research partners, lay collaborators, and patient participants. The preliminary PROM-OPAC integrated seven novel ‘Autonomy’ items and the best available ‘Function’ instrument, EQ-5D-5L. PROM-OPAC is undergoing field-testing and validation.

## A8 ForMi—Person-centred planning and outcomes recording App

### *^1^Roger Rowett, ^2^Idris Baker

#### ^1^Here2there.me Ltd, Denbigh, United Kingdom. ^2^Swansea Bay University Health Board, Swansea, United Kingdom

Health in Wales states^1^ that ‘it is patients themselves who are best placed to judge how they feel’. We therefore need ways to ask them to ‘assess how they feel, from their own perspective’ against a standardised outcome framework. This presentation will report on a research and development project to address this need, funded by Welsh Government through the Small Business Research Initiative (SBRI) and led by Here2there.me Ltd (H2t). It offers a novel way to blend the setting and recording of personal goals with the use of standardised outcomes frameworks. H2t has developed a person-centred planning and outcomes recording tool called ‘ForMi’. This recognises that outcomes are part of a larger cycle that starts with a person-centred plan. The individual (a patient in this case) has as much ownership of the plan as possible. The tool has been piloted within 9 sites across Wales. The individual (with support) agrees a strength-based profile, a set of goals (in their own words) and a Circle of Support. The Circle could include people from social services, health, education, friends and family: whoever is best placed to help them achieve the goals most important TO them, as well as for them, and record these as outcomes. The agreed goals can be tagged against any standardised outcomes framework. This allows for a fully individualised approach to outcome setting, whilst still enabling standardised PROMS reporting. The goals are ‘rated’ by the person and a key worker on a 0–10 scale (as recommended by Welsh Government^2^) at the beginning and end of the intervention or treatment. Depending on the length of the intervention, there may also be intermediate ratings of progress. The system is managed online via a Control Panel. Users are also able to use an App to record their ‘story’ of achievement against their goals. This unique functionality is similar to many social media platforms, allowing the individual and their Circle of Support to view and upload comments and pictures to support their progress against their goals. This facilitates joined-up working by multi-disciplinary teams and provides crucial evidence to support achievement against outcomes, beyond the subjective opinions of individuals and professionals. We will present experience from pilot projects where the system has been used and will consider other settings where its use could be studied.

^1^http://www.wales.nhs.uk/promspremsandefficiencyprogramme (accessed 10.3.2022).

^2^https://gov.wales/recording-progress-personal-outcomes-care-plan-guidance (accessed 11.3.2022).

## Abstract Session 1C

## A9 PROMs: Coming of age in Lymphoedema Services in Wales

### *^1^Marie Gabe-Walters, ^1^Melanie Thomas

#### ^1^Lymphoedema Wales, Swansea, United Kingdom

Introduction: A Lymphoedema-specific Patient Reported Outcome Measure (LYMPROM©) for adults has been completed online in three Health Board Lymphoedema Services since late 2020. Using an automated digital platform, integrated with the local patient management system, LYMPROM© is shared when a patient is referred to their local service and two-weeks before any planned follow-up. The 13-item LYMPROM© reminds patients to report their impact of lymphoedema from zero to 10, where 10 represents extreme impact, with a free-text section at the end. As part of the Value Based Healthcare (VBHC) initiative, Lymphoedema Wales has collaborated with Digital Health and Care Wales and the Welsh Value in Health Centre to develop a LYMPROM© dashboard to view aggregate data. Aims: To examine the implementation and use of LYMPROM© using an automated platform. Methods: Data are descriptively reported alongside user feedback. Results: Almost 6000 (5754) LYMPROM© forms have been completed online. Based on annual data (January 2021–2022), the overall response rate is 38.05% (4851/12750) with patients typically responding within four days (average). Patients have reported that LYMPROM© helps them say what matters and focuses their appointment. However, some concern for the legitimacy of the text/email notification was initially signposted, with ongoing challenges to complete digitally. Feedback from therapists indicates a need to engage with patients to improve their awareness of the purpose of PROMs. Therapists have adapted to using a digital platform to view LYMPROM© in their daily workings. At the patient level, the platform provides oversight of patient reported outcomes over time; helping to focus the priorities of assessment and patient care. Based on aggregate data, shopping for clothes / shoes (mean = 6.03, SD 3.64), body image (mean = 5.70 SD 3.47), intimacy / desirability (mean = 5.65 SD 3.79) and heaviness (mean = 5.58 SD 3.01) are the biggest challenges for patients with lymphoedema. To further this work, the Lymphoedema Wales LYMPROM© dashboard was launched to key stakeholders in March 2022 as part of VBHC. Conclusions: Automated digital access is minimising the effort in sending LYMPROM© and is helping therapists plan care in line with what matters to patients. However challenges remain with engagement and digital access / literacy. Resources have been developed to support the switch to digital collection for patients, with training provided for staff. The dashboard provides the opportunity for service providers and planners to review LYMPROM© data. Subsequent review and phases of dashboard development will help maximise the benefits of PROM-led care.

## A10 True Colours online mood monitoring in the Bipolar Disorder Research Network (BDRN) research programme: Challenges, benefits and importance of personalisation

### *^1^Katherine Gordon-Smith, ^2^Kate Saunders, Julia Savage, ^3^Ian Jones, ^1^Lisa Jones

#### ^1^University of Worcester, Worcester, United Kingdom. ^2^University of Oxford, Oxford, United Kingdom. ^3^Cardiff University, Cardiff, United Kingdom. ^4^University of Worcester, Worcester, United Kingdom

Bipolar disorder (BD) is a common mental health disorder which affects approximately 2% of the population and is associated with significant morbidity and mortality. It is characterised by episodes of depression and hypomania/mania which vary in severity both between and among individuals. These mood episodes can cause significant problems in everyday life including relationships and work, and many people with BD report ongoing symptoms outside of mood episodes. In recent years there has been an emergence of an increasing number of electronic mood monitoring tools designed for individuals with BD in both clinical and research settings. These tools have predominately employed predefined symptom-based questions to monitor mood at varying time intervals usually ranging from multiple times a day to weekly. We have introduced the True Colours weekly electronic mood monitoring tool into our large-scale UK-wide BDRN research programme. The BDRN True Colours system sends participants weekly email prompts to complete two online self-report questionnaires which measure presence and severity of depressive and hypomanic/manic symptoms over the preceding week. The tool also allows participants to view their longitudinal symptom scores graphically outside of the clinical environment. To date over 1200 BDRN participants have joined True Colours, and of those who have had the opportunity, 50% have engaged for at least 52 weeks with < 10% engaging for less than one month. Reported patient benefits include tracking moods, spotting trends and triggers, communicating experiences to others, and aiding self-management. Early participants reported that the mood questionnaires alone were not capturing fully their experiences of living with BD. In response to this feedback we added the option for participants to create their own personalised questions to monitor, for example, sleep, physical activity levels, physical health, and mood instability. Thematic analysis of the content of these questions revealed many aspects of BD important to patients in relation to longitudinal monitoring that extended well beyond mood symptoms. Our findings highlight the importance of individualised measures in helping to capture the natural trajectory of BD from the patient perspective. Additional symptoms and aspects of life than those useful diagnostically for BD may be more important for individuals themselves to monitor and have more meaning in capturing their own experience of BD. Future research into the relationships between longitudinally measured patient priority aspects of BD, mood symptoms and long-term outcomes are warranted. These findings may aid the development of clinically effective real-time online personalised self-management tools.

## A11 published 10.1001/jama.2022.6421

## A12 Understanding PROMs systems to help fit the square “routinely collected PROMs” peg to the circular “healthcare question” hole

### *^1^Robert Palmer

#### Cedar Health Technology Research Centre, Cardiff and Vale UHB, Cardiff, United Kingdom

PROMs analysis can be carried out prospectively or retrospectively. Prospective studies have healthcare questions ready before designing the PROMs collection system. Referring to the well-known analogy: a circular “PROMs” peg has been deliberately collected to fit the circular “question” hole. With the rising adoption of routinely collecting PROMs in standard care, many analyses are retrospective. Questions are drawn-up after data is collected, and secondary data often used to answer questions. The square “routinely-collected PROMs” peg therefore, needs moulding to fit the circular “question” hole. Routinely collecting PROMs yields disordered datasets, with patients often completing at inconsistent times. This is often a characteristic of the collection system. Whilst paper and electronic-based systems have their own advantages and disadvantages mainly concerning completion rates, it’s the system as a whole that affects how datasets look. Systems can be represented on a scale between being “rigid” and “flexible”. Rigid systems have greater control over PROMs collection. One PROM can be completed per patient per invite, and submitted within a short time period. An example is inviting patients to complete a PROM during their appointment visit. Patients can only complete one PROM while they’re in the building on that day. Flexible systems allow patients to complete whenever and as often as they like, producing larger datasets. Examples include sending patients a link to an e-PROM. Once submitted patients can re-submit additional PROMs at any time using the same link. In prospective studies such as clinical trials, collection is usually on the rigid end of the scale. Routine collection systems however are rarely that rigid. Most platforms collect remotely, and patients don’t always reply straight away. NHS Wales’ National PROMs platform is towards the flexible end, as patients can complete new PROMs any time if they feel their health changing. Implementation of systems using platforms like Amplitude Clinical Outcomes, DrDoctor and Patient Knows Best also lie somewhere on this scale. Differences between them include: When patients are invited to complete PROMs forms; When patients can complete forms; When patients can submit forms; Number of forms patients can complete. Graphical illustrations of data from systems lying on the rigid-flexible scale will show how different systems affect datasets, and a solution to dealing with these system-specific differences will be presented. These often forgotten-about characteristics should be considered when rounding the square peg of routinely collected PROMs.

## Abstract Session 2A

## A13 Patient Reported Outcome Measures for Rheumatoid Arthritis Disease Activity: using Rasch measurement theory to achieve more meaningful measurement

### *^1^Tim Pickles, ^2^Mike Horton, ^3^Karl Bang Christensen, ^4^Rhiannon Phillips, ^5^Ernest Choy

#### ^1^Cardiff University, Cardiff, United Kingdom. ^2^University of Leeds, Leeds, United Kingdom. ^3^University of Copenhagen, Copenhagen, Denmark. ^4^Cardiff Metropolitan University, Cardiff, United Kingdom

Disease Activity (DA) monitoring is a standard of care in Rheumatoid Arthritis (RA), and there is demand for achieving this through Patient Reported Outcome Measures (PROMs). However, a suitable PROM would need to display acceptable measurement properties. A systematic review of PROMs for RA DA following COSMIN guidelines demonstrated a lack of sufficient evidence for content validity for the 10 existing PROMS and thus concluded that none can be recommended for use. The aim of this study is to use Rasch measurement theory to develop a valid item pool for measurement of DA in RA, moving towards future implementation of a computer adaptive testing (CAT) system. Paper questionnaires were sent to people aged 18 or over with RA from four South Wales University Health Boards between September 2020 and November 2021. The questionnaire included 268 individual RA DA items extracted from the 10 PROMs identified by the systematic review, another PROM not included in the systematic review, a foot-specific PROM, two flare PROMs and a non-measurement group of items. Further items suggested by patients and PPI were also incorporated, including a Pain Activity Scale, discomfort when walking, when standing, and when exercising, plus fear of falling when walking. Demographics were collected, and respondents were given the option to be invited to take part in cognitive interviews and a dissemination event. We collected a dataset of n = 677 in order to develop the item pool. Psychometric properties of all PROMs will be assessed by Rasch measurement theory analyses, which provides results on targeting and item locations, fit to the Rasch model, reliability, local dependency, uni-dimensionality and item threshold ordering. Further analyses will include Mann–Whitney U tests and area under the receiver operating characteristic curve to evaluate if the PROM is able to discriminate between flare and non-flare populations, Spearman’s ρ, Cronbach’s α and confirmatory factor analyses (Χ2 test of fit, RMSEA, CFI, TLI, SRMR). We will go through an iterative process taking items from various PROMs to create an item pool that can be shown to satisfy all necessary aspects. Additionally, a purposive sample of respondents will take part in cognitive interviews to assess validity of items in terms of content and response processes. A CAT will be built on the locations of items in the item pool. This would personalise the PROM and optimise its potential for use in routine clinical practice.

## A14 Developing a roadmap towards national collection of electronic patient-reported outcomes for people with chronic kidney disease in the UK

### *^1,2^Helen V Chadwick, ^1,2^Angelo Ercia, ^3^Sarah E Knowles, ^1,2^Sabine N van der Veer

#### ^1^Centre for Health Informatics, Division of Informatics, Imaging and Data Sciences, Faculty of Biology, Medicine and Health, Manchester. ^2^Academic Health Science Centre, The University of Manchester, Manchester, United Kingdom. ^3^Centre for Reviews & Dissemination, University of York, York, United Kingdom

Aim: Develop a roadmap for the next 10 years that describes how to establish a national system for collecting electronic patient-reported outcomes (ePROs) for people with kidney disease in the UK. Ultimately, this will enable people with kidney disease to be more involved in their own care and improve their outcomes. Methods: We explored views of key stakeholders on what was needed for establishing a national ePRO system for kidney care in the UK. Key stakeholder groups included kidney patients, healthcare professionals, commissioners, and facilitating organisations (e.g., national audit and service improvement organisations, industry partners, academia). We conducted semi-structured interviews with 18 stakeholder representatives to elicit their perspectives on the topic. In addition, we organised five parallel focus groups as part an online stakeholder event with a total of 58 participants. Focus group topics included: measurement instrument, technology and infrastructure, implementation, ePRO-generated inequalities, and multimorbidity. The research team analysed and synthesised all data thematically. With input from stakeholder representatives, they translated themes into recommendations for how to achieve national collection of ePROs in kidney disease. Results & conclusions: Preliminary analyses from the semi-structured interviews and focus group discussions suggested that not all stakeholders were aware of or had shared views on the potential benefits of ePROs. They indicated that national ePRO collection required support from local healthcare professionals at all organisational levels, while ensuring that solutions for data collection could be adapted to local contexts and patient groups. Stakeholders also suggested that –in addition to evidence of clinical effectiveness—examples of the usability and feasibility of data collection would support the case for a national ePRO system. Lastly, they recommended harnessing existing regional renal service improvement networks as a suitable infrastructure for scaling up ePRO collection, and developing national guidance to guide this wider roll-out. We are currently undertaking the final step, where we present the preliminary findings to the stakeholder representatives as a starting point for co-developing a set of key recommendations for how to achieve national collection of ePROs in kidney disease in the next 10 years.

## A15 Measuring bereavement support needs in people bereaved during Covid-19; the adaptation and development of a bereavement support needs scale

### ^*1^Emily Harrop, ^2^Damian Farnell, ^1^Mirella Longo, ^1^Silvia Goss, ^1^Stephanie Sivell, ^1^Kathy Seddon, ^1^Annmarie Nelson, ^1^Anthony Byrne, ^3^Lucy Selman

#### ^1^Cardiff University, Marie Curie Palliative Care Research Centre, School of Medicine, Cardiff, United Kingdom. ^2^Cardiff University, School of Dentistry, Cardiff, United Kingdom. ^3^University of Bristol, Palliative and End of Life Care Research Group, Bristol Medical School, Bristol, United Kingdom

Background: Using consensus methodologies, we previously identified two core outcomes and associated dimensions for designing and evaluating bereavement support interventions in palliative care: ‘Ability to cope with grief’ and ‘quality of life and mental wellbeing’ (Harrop 2020 10.1186/s12904-020-0532-4). In a subsequent research study investigating bereavement experiences during COVID-19 (www.covidbereavement.com) we adapted these outcome dimensions to create a 13-domain scale assessing bereavement support need, which we discuss here. Methods: The support needs scale includes emotional (n = 10) and practical (n = 3) domains. The scale (and survey) was piloted with 16 members of the public with bereavement experiences. Using interim results involving 532 survey participants, two subscales (emotional support and practical support) were found via exploratory factor analysis. Cronbach’s α were 0.79 and 0.95 for the practical and emotional subscales, respectively, and 0.94 for all items, indicating high levels of reliability/internal consistency. Subscale scores are found by determining the mean across all items in a given subscale. The overall mean is evaluated over all 13 items. We interpret results for both subscale scores and the overall mean score via: 1 = no support needed; 3 = moderate level of support needed; 5 = high level of support needed. Results: This support needs scale enabled us to identify domains where support need was highest (e.g. dealing with my feelings about how my loved one died, expressing my feelings and feeling understood by others) and calculate overall scores for the scale and two sub-scales. For the practical subscale, mean = 2.41 (95% CI = 2.34 to 2.50), indicating little to moderate level of practical support needed. For the emotional subscale, mean = 3.33 (95% CI = 3.25 to 3.41), indicating moderate level of emotional support needed. Results for the emotional subscale were significantly (P < 0.001) higher than for the practical subscale. The scale was used to identify factors associated with higher levels of support need in our cohort of bereaved participants (e.g. relationship with deceased, social isolation and loneliness). Conclusion: This support needs scale represents a novel and pragmatic adaptation of an outcome set which was originally intended for use in the design and evaluation of bereavement interventions. It has practical benefits for improving bereavement support provision by both highlighting the specific domains where support needs are highest, and as a tool for identifying potential variations in support need across demographic and clinical groups and tailoring support accordingly.

## A16 The Scottish Cancer PROMs Advisory Group: A ‘once for Scotland’ strategy for the implementation of PROMs

### *^1^Emma Dunlop, ^2^Kelly Baillie, ^2^Jennifer Laskey, ^3^Debbie Provan, ^4^Peter Hall, ^1^Marion Bennie

#### ^1^University of Strathclyde, Glasgow, United Kingdom. ^2^NHS Greater Glasgow & Clyde, Glasgow, United Kingdom. ^3^Scottish Government, Edinburgh, United Kingdom. ^4^University of Edinburgh, Edinburgh, United Kingdom

Background: There is increasing motivation to embed patient reported outcome measures (PROMs) into routine cancer care to monitor the real-world impact of cancer treatment on quality of life. Routine PROMs collection/use could: inform shared treatment decisions; enhance patient care; provide population-level treatment outcome evaluation, and inform clinical guidance and care pathways; and support the health technology assessment (HTA) process. Through Scotland’s Innovative Healthcare Delivery Programme, PROMs are firmly positioned within the Scottish Government’s cancer strategy. The Cancer Medicines Outcomes Programme (CMOP) is a national collaboration between Scottish NHS Boards and the University of Strathclyde. CMOP aims to develop a robust and reliable process to understand the effectiveness and safety of cancer medicines in routine care in Scotland. One objective is to provide strategic leadership in implementing cancer medicines PROMs in clinical practice. After conducting some early PROMs studies in Phase 1, CMOP recognised the need for more strategic leadership in PROMs in clinical practice, and the need for a more cohesive approach across Scotland. Our Approach: The Scottish Cancer PROMs Advisory Group & Forum: The Scottish Cancer PROMs Advisory Group (SC PROMs AG) was established in 2021. The goal is to have a “once for Scotland” approach to implementing and adopting a set of core principles that include PROMs items/tools plus guidelines for how PROMs collection can be integrated into existing care pathways and digital systems. The group engages clinicians, charities, researchers, eHealth and other stakeholders embarking upon PROMs. The SC PROMs AG has also formed a Scottish Cancer PROMs Forum—an open collaborative space for stakeholders, (including patients, members of the public and digital companies). The first Forum meeting (March 2022) had in excess of 170 people registered to attend, demonstrating support for our collaborative approach and interest in cancer PROMs and PROMs generally in Scotland. Our Goals: The aim of the SC PROMs AG is to enhance and inform current and new PROMs projects, identify opportunities for collaborative research and maximise opportunities for shared learning from PROMs use. The Group will guide clinical practice, research, strategy and policies relevant to the collection and use of PROMs with cancer patients. Informing the SC PROMs AG will be the Forum discussions and shared learning across current PROMs research and work streams, potentially minimising duplication of effort and patient population burden in the testing of PROMs digital tools.

Abstract Session 2B.

## A17 Patient reported outcome assessment must be inclusive and equitable

### *^1^Melanie J Calvert, ^1^Samantha Cruz, ^1^Ameeta Retzer, ^1^Sarah E Hughes, ^2^Lisa Campbell, ^3^Barbara Molony-Oates, ^1^Olalekan Lee Aiyegbusi, ^4^Angela M Stover, ^5^Roger Wilson, ^1^Chistel McMullan, ^1^Nicola E Anderson, ^1^Grace M Turner, ^6^Elin Haf Davies, ^1^Rav Verdi, ^7^Galina Velikova, ^8^Paul Kamudoni, ^1^Syed Muslim, ^9^Adrian Gheorghe, ^2^Daniel O’Connor, ^1^Xiaoxuan Liu, ^10^Albert W Wu, ^1^Alastair K Denniston

#### ^1^University of Birmingham, Birmingham, United Kingdom. ^2^Medicines and Healthcare products Regulatory Agency (MHRA), London, United Kingdom. ^3^Health Research Authority, London, United Kingdom. ^4^University of North Carolina, North Carolina, USA. ^5^NCRI Consumer Forum National Cancer Research Institute, London, United Kingdom. ^6^Aparito Limited, Wrexham, United Kingdom. ^7^University of Leeds, Leeds, United Kingdom.^8^Healthcare Business of Merck KGaA, Darmstadt, Germany. ^9^Imperial College London, London, United Kingdom. ^10^Johns Hopkins Bloomberg School of Public Health, Baltimore, United Kingdom

Patient-reported outcomes (PROs) are collected in clinical trials to provide valuable evidence on the risks and benefits of treatment and in routine clinical practice to support patient-centered care. To increase the positive impact of PRO data and to avoid the unintended consequence of increasing health disparities, we need to consider the needs of under-served groups and identify approaches to ensure greater equality, diversity and inclusion (EDI). To propose actions to promote representation of under-served groups in the collection of PRO data. A rapid literature review to identify and summarise key publications and consultation with international stakeholders (n = 20) and patient partners (n = 2) to 1) identify barriers to EDI and 2) formulate key actions to promote representation of under-served groups in the collection of PRO data. Several challenges to EDI were identified. These included a lack of valid and reliable PRO measures that have been co-developed with, or are relevant to, the target population. PRO measures developed with limited patient input risk omission of key concepts of importance to under-served groups. This is particularly true if these groups are excluded from concept elicitation due to communication barriers arising from learning disabilities, low literacy, or digital exclusion. Failure by trialists and clinicians to use translated and culturally validated PROs threatens to increase racial and ethnic disparities through exclusion of minority ethnic groups from PRO reporting. Lack of culturally appropriate and linguistically validated measures limits the use of PROs within low- and middle-income countries. To promote the representation and participation of under-served population in PROs several actions were proposed: 1) widen participation by ensuring individuals involved in PRO co-development are representative of the target population; 2) be mindful of the clinical characteristics of the disease when designing or selecting a PRO to minimise barriers to completion; 3) acknowledge cultural values through the use of translations; 4) providing accommodations to ensure individuals are able to complete a PRO regardless of ability to read, write and problem solve; 5) consider ways to promote digital inclusion; and 6) engage regulators in EDI discussions early in the drug development life cycle. PRO data needs to reflect the diversity of modern society. Implementation of specific actions to address EDI, both in trials and routine care, can promote representation of under-served groups, reduce health disparities, and result in the collection of meaningful PRO data for the benefit of all.

## A18 A systematic review of quality of life and health-related quality of life as outcome measures in substance and behavioural addictions

### *^1^Andrew Dyer, ^2^Jan R. Boehnke, ^1^David Curran, ^1^Katie McGrath, ^1^Paul Toner

#### ^1^Centre for Improving Health-Related Quality of Life, School of Psychology, Queen's University Belfast, Belfast, United Kingdom. ^2^School of Health Sciences, University of Dundee, Dundee, United Kingdom

The assessment and treatment of substance-related and addictive disorders can benefit from a holistic consideration of an individual’s quality of life (QoL), however, there remains uncertainty over how the construct is operationalised as an outcome measure. The current systematic review aimed to identify all the QoL and health-related quality of life (HRQoL) instruments adopted as outcome measures in addiction research and map the conceptualised domains. Available psychometric evidence supporting their use was also summarised. A systematic search of three electronic databases and a specialised assessment library was conducted for studies utilising a QoL or HRQoL instrument as an outcome measure. Participants using or taking part at risky levels and above assessed with a valid measure were included. Preferred Reporting Items for Systematic Reviews and Meta-Analyses (PRISMA) guidance was followed and included outcome instruments were appraised using mixed-methods. Validation studies were assessed for their risk of bias based on the Consensus-based Standards for the selection of health Measurement Instruments (COSMIN) risk of bias tool. Two hundred and thirty articles containing 258 discrete studies were included. Forty-seven outcome instruments were used: 28 assessing QoL in 141 studies; 19 assessing HRQoL in 117 studies. The WHOQOL-BREF was the most popular instrument utilised in 73 studies. Content analysis identified 39 unique domains of QoL. Eighteen articles comprising 20 validation studies evaluated the psychometric properties of 11 outcome measures. No instrument was assessed for the same parameter in 5 or more studies for meta-analytic pooling purposes. The ALQoLS, ALQoL-9, Q-LES-Q-SF, SF-36, and WHOQoL-BREF all produced multiple, promising internal consistency statistics (Cronbach’s α = 0.75–0.97), but with varying degrees of methodological quality. Other parameters of reliability and validity are also reported. It is clear many QoL and HRQoL instruments have been utilised in the field. However, a significant portion of studies applied a small number of popular instruments for which there is minimal high-quality validation evidence provided to support their use with populations at risk of addiction. There is a need for more rigorous primary studies with validation evidence presented for the appropriateness of the QoL or HRQoL assessment instrument chosen.

## A19 Development of a conceptual framework to reflect what is important to adults after a lower limb reconstruction: PROLLIT

### *^1^Heather Leggett, ^1^Arabella Scantlebury, ^1^Catherine Hewitt, ^2^Hemant Sharma, ^1^Catriona McDaid

#### ^1^The University of York, York, United Kingdom. ^2^Hull University Teaching Hospitals NHS Trust, Hull, United Kingdom

Patient reported outcome measures (PROMs) are used to understand the impact of lower limb reconstruction on patient’s quality of life (QOL). Existing measures have not been developed to specifically capture patient experiences amongst adults with lower limb conditions that require reconstructive surgery. This research aimed to develop a conceptual framework to reflect what is important to patients requiring, undergoing or after undergoing reconstructive surgery and ascertain whether these are currently captured in PROMS used for this group of patients. Our population of interest was people requiring, undergoing or after undergoing reconstructive surgery due to trauma, malunion, nonunion, infection and congenital issues treated by internal or external fixation. Our research entailed three steps: Step A: Qualitative Evidence Synthesis: MEDLINE, Embase, PsychINFO and Cinahl were searched from inception until November 2020. Thematic synthesis was undertaken on 9 included studies and 8 domains were identified as important to patients: Pain, Identity, Income, Daily lifestyle and functioning, Emotional well-being, Support, Ability to adapt and adjust, Ability to move forwards. These findings led to the development of a preliminary conceptual framework Step B: Qualitative study: Interviews with 32 patients and 22 orthopaedic staff (surgeons, methodologists and patient contributors) were undertaken between November 2020 and June 2021 in England. The 8 domains from the preliminary conceptual framework were used as a framework around which to code the interviews. These findings led to the refinement of the conceptual framework. Step C: Interdisciplinary meetings: The research team ran three meetings with members of the advisory panel: orthopaedic surgeons, methodologists and PPIE members and further refined the conceptual framework. Six domains important to patients were included in the final conceptual framework: Pain, Perception of self, Work and finances, Daily lifestyle and functioning, Emotional well-being and Support. The first five relate to important outcomes for patients. These domains are all inter-related and their importance to patients changed as they recovered. The final domain- Support (from the hospital, physiotherapists and family/friends and feeling informed about the next steps in their recovery)—was vital to patients and lessened the negative impact of the other domains on their quality of life. This research has identified 6 areas that are important to patients during or after a lower limb reconstruction. The next step in this research is to ascertain whether current PROMs used with this group of patients adequately capture these areas of importance.

## A20 published 10.1007/s40271-021-00555-7

## Abstract session 2C

## A21 published 10.1002/jmv.27878

## A22 PROMs-based Key Performance Indicators (KPIs) to evaluate waiting list prioritisation schemes against prudent healthcare principles

### ^1^Robert Palmer, *^2^Geraint Palmer

#### ^1^Cedar Health Technology Research Centre, Cardiff and Vale UHB, United Kingdom. ^2^Cardiff University, Cardiff, United Kingdom

Waiting list prioritisation schemes that attempt to treat those with greater need first following prudent healthcare principles can be simulated to predict performance. As it’s difficult to characterise patient need with a single metric, common KPIs focus on average waiting times. These do not tell us how population health is affected, nor if those in greater need are treated sooner. As patients are best-placed to assess their health, we propose using discrete event simulation (DES) and three KPIs using PROMs as indicators of patient need to assess how prioritisation schemes help achieve prudent healthcare. DES models queueing systems by repeatedly adding random patients to a virtual queue, ordering it according to prioritisation rules, and removing patients when treatment slots become available. KPI 1 is the correlation between pre-surgery PROM score and waiting time. Positive correlations indicate that those in greater need are treated first. KPIs 2 and 3 are interpreted from PROM score adjusted life-years (PSALYs), which consider condition-specific PROMs (i.e. Oxford Hip Score) and are more sensitive to specific (i.e. hip) symptom changes than QALYs. We define PSALYs to include symptom severity and quantity of life lived: PSALY = PROMs Score x Time. Two pre-surgery PSALYs are calculated per patient; with prioritisation and without (i.e. first-in first-out (FIFO)), and differences calculated. Queue-jumped patients have positive differences suggesting less pre-surgery symptom burden compared to FIFO, while those pushed back have negative values. KPI 2 is the mean difference in pre-surgery PSALY between prioritisation and FIFO of all patients, indicating overall pre-surgery symptom burden across the population. KPI 3 is the percentage of patients with positive PSALY differences, i.e. those benefitting from the scheme compared to FIFO. To apply our KPIs, we used NHS Digital hip replacement PROMs datasets to simulate four arbitrary schemes. Patients were prioritised according to presence of diabetes (scheme 1), arthritis (2), number of co-morbidities (3) and pre-surgery EQ-VAS (4). KPI 1 showed that schemes 2, 3 and 4 treated those with greater need first. KPIs 2 and 3 showed that while scheme 1 slightly improved overall pre-surgery burden, under a tenth of patients actually benefitted. Schemes 2, 3 and 4 yielded higher overall burden, but the majority of patients benefitted, suggesting significantly hindered outlying patients. This work shows that condition-specific PROM scores can be used as a convenient and sensitive single indicator of patient need to evaluate prioritisation schemes against prudent healthcare principles.

## A23 Designing Pre-Registration Curricula to Routinise the Incorporation of Patient-Reported Outcomes in Healthcare Professional Practice

### *^1^Angela Wolff, ^2^Lisa Edwards, ^1^Deborah Gibson, ^2^Heidi Boyd

#### ^1^Trinity Western University, Langley, Canada. ^2^University of Bradford, Bradford, United Kingdom

An essential component of healthcare professional (HCP) practice is the collection of clinically relevant information from patients to better understand and address their health concerns. This is commonly referred to as the gathering of subjective and objective information. A more recent practice has been the addition of patient-reported outcome and experience measures (PROMs/PREMs, respectively). Although the use of these tools in practice are becoming more prevalent, often pre-registration curricula for healthcare professionals does not include relevant education regarding PROMs and PREMs. The aim of this knowledge-translation presentation is to provide the knowledge, tools, and resources to design curricular, theory/content and learning activities for PROM/PREMs education at undergraduate level. This presentation is based on a newly created resource guide that focusses on the needs of HCPs and factors that influence PROM/PREM adoption in practice. Using a mixed method design, this guide is based on (a) evidence from a systematic review, qualitative HCP interviews, and stakeholder consensus-building, and (b) implementation science frameworks. We discuss the development and delivery of effective PROM/PREMs education in pre-registration curricula with a focus on knowledge development and skill acquisition to inform HCPs clinical reasoning, judgement, and subsequent course of action. Using backward design for educational experiences, exemplars from registered nurse and physiotherapy pre-registration programs are included. In summary, the implementation of PROM / PREMs into practice requires careful consideration of ways to integrate these tools into curricula. Embedding PROM/PREMs into pre-registration education could facilitate their inclusion in the routine practice of post-registration, novice clinicians. Facilitating HCP adoption of PROM/PREMs to include patients’ voice in their care is a complex behaviour change that can start by preparing the next generation of HCP. Using the proposed, resource guide can facilitate this process to address the needs of HCPs and specific barriers to PROM/PREM implementation.

## A24 The Association of Baseline Score and Minimal Clinically Important Difference in Hip Replacements – An Exploration Using Item Response Theory and Interval Scale Methods

### *^1^Jonathan Evans, ^1^Alex Matthews, ^1,2^Jose Valderas

#### ^1^University of Exeter, Exeter, United Kingdom. Yong Loo Lin School of Medicine, Singapore, Singapore

Background: The minimal clinically important difference (MCID) defines the smallest difference in a patient reported outcome measure that patients perceive as beneficial. Although numerous methods are used to derive this value, classically a single value across the scale is employed. This value does not take into account any variability that may be associated with the baseline score. This study aims to assess the impact of baseline score on MCID, and explores whether a score derived at a trait level using item response theory improves the accuracy of the MCID estimation. Methods: The MCID of the Oxford Hip Score was derived from data on 149,055 patients who received a primary hip replacement. The anchor ‘a little better’ at 6-months on the global change score defined minimal improvement. The MCID was calculated for the whole cohort and baseline score subgroups using interval scale and IRT derived scores. The sensitivity (sens), specificity (spec), area under the curve (AUC) of the receiver operating characteristic curve, positive (ppv) and negative (npv) predictive value and Odds ratio (OR) were calculated using the global change score as the gold standard. Results: The MCID for the without baseline calibration was 12.69 (interval scale) and 1.39 (IRT scale). With baseline calibration, the MCID ranged from 3.51 – 17.29 (interval scale) and 0.56 – 2.14 (IRT scale). The sens, spec, ppv and npv were similar for the MCID derived from interval and IRT scales. However, if the MCID was defined as a function of baseline score, the sens, spec, ppv and npv were consistently higher. For the interval scale, ROC AUC was 0.6 (95%CI 0.6 – 0.61) and OR 24.9 (95% CI 24.4 – 26.5) with no calibration vs AUC 0.63 (95%CI 0.63—0.63) and OR 37.7 (95%CI 35.3 – 40.2) with baseline calibration. For IRT scales AUC 0.59 (95%CI 0.59 – 0.6) and OR 22.2 (95%CI 20.9 – 23.1) vs AUC 0.63 (95%CI 0.62 – 0.63) and OR 31.8 (95%CI 31.8 – 36.0). Conclusion: This study highlights the need to consider the MCID as a function of the baseline score. In PROMs used ubiquitously to assess the effect of an intervention in trials and longitudinal cohort analysis, not doing so risks the introduction of error. The use of IRT derived scoring did not improve the accuracy of the estimation.

## Abstracts for Poster Presentation

## A25 Convergent validity of EQ-5D with core outcomes in dementia: a systematic review

### ^1^Hannah Hussain, ^1^Anju Keetharuth, ^1^Donna Rowen, ^1^Allan Wailoo

#### ^1^University of Sheffield, Sheffield, United Kingdom

Objectives: To explore the convergent validity of EQ-5D (total score and dimensions) with core outcomes in dementia by systematically reviewing the literature to understand these empirical relationships, and how they may be impacted by EQ-5D rater-type. Methods: To identify articles relevant to the convergent validity of both the three-level and five-level versions of EQ-5D with core dementia outcomes, three electronic databases were searched in April 2021. Pre-defined exclusion and inclusion criteria were applied upon screening of the records. A purposefully developed data extraction form was used to capture the relevant data, and a narrative synthesis was adopted. Results: The search strategy retrieved 236 unique records, of which 29 met the inclusion criteria for the review. Twelve different core outcome instruments were used to capture the dementia outcomes: cognition, function, and behaviour/mood across the studies, of which the MMSE was the most dominant tool. The majority of the studies used EQ-5D-3L (n = 24). EQ-5D had a clearer, stronger relationship with the measures of function and behaviour/mood, showing little evidence of association with cognition. EQ-5D dimensions exhibited associations with the appropriate corresponding clinical outcomes, for which the relationships were stronger with proxy-EQ-5D than for self-rated EQ-5D. Conclusion: Measuring health-rated quality of life (HRQoL) in dementia populations is a complex issue, particularly when considering balancing the challenges associated with both self and proxy rating. While EQ-5D-3L shows good convergent validity with dementia outcome measures and captures the key symptoms relevant to dementia HRQoL, there is a need for more evidence on EQ-5D-5L. Future research should focus on how to address the little evidence of association of EQ-5D with cognition.

## A26 The need for Nurse Researchers and how their research can be instrumental in embedding a positive research culture into practice

### ^1^Emma Williams

#### ^1^Cardiff and Vale University Health Board, Cardiff, United Kingdom

There are around 165,000 cancer deaths every year, accounting to more than a quarter of all deaths (Cancer Research UK 2020). Treatments are rapidly progressing and people are experiencing cancer not just as life limiting, but often as a life changing condition (Foster 2019 10.1186/s13014-019-1222-3). Understanding the quality of life of patients within our care is important and will help us to guide and inform any subsequent interventions. Quality of life is essential when considering a person's integrated feelings (Buting 2020). As givers of CART products within the UK we are commissioned to deliver care in a patient centred way via a values-based healthcare approach and need to understand more about the quality of life of patients. There is a paucity of data with regards to a patient’s quality of life throughout having CART therapy and a lack of European data into this novel area of treatment. CART-QUOL is a study designed by Emma Williams (Nurse Chief Investigator) and aims to collect quality of life assessments at regular time points, before during and after treatment with CART treatment, thus exploring the real-world experiences of patients who receive this novel cellular therapy. This nurse led study is done in collaboration with the South Wales Blood and Bone Marrow Transplant Programme (SWBMT) and is the first Centre in Wales to offer CART therapy. Cellular therapies and regenerative medicine are research themes of specialist interest within the SWBMT programme. The study has been developed with a view to encouraging more nurses to take on research as currently there is a lack of nurse researchers throughout the literature (Higgins 2010 10.1016/j.nedt.2009.10.017, Albert 2016 10.1097/NUR.0000000000000236, Watmough 2010 10.12968/bjca.2010.5.8.71939, Loke 2012 10.1016/j.nedt.2012.09.006). Nurse should feel empowered to take on research projects and are often best placed as they are at the forefront of care. The study was set up and opened at the height of the Covid pandemic, and is a positive example of how nursing research can be done despite immense pressures within the NHS. It aims to motivate others to take a proactive approach to research. Nursing research can and must be performed alongside all other research and if done to a high standard and in collaboration with a supportive team, can enhance and give greater depth to a research portfolio. Patient reported outcome measures are vital in practice and nurses can contribute significantly to this ongoing agenda.

## A27 A study protocol to develop the domains of an observational well-being scale (WEBS) for non-verbal children and young people with cerebral palsy from using the Innowalk

### ^1^Dawn Pickering, ^1^Tim Pickles, Ted Shiress

#### ^1^Cardiff University, Cardiff, United Kingdom

Cerebral palsy (CP) is a group of permanent disorders of the development of movement and posture often accompanied by disturbances of communication and behaviour. For those with more severe physical disabilities, their ability to participate in physical activities is limited, which includes those with walking limitations. It is known that adults with CP are prone to early development of chronic diseases such as a cardiovascular disease. Increasing physical activity levels improves well-being across the general population, including children without disabilities. Whether this is so for those children who have mobility limitations and cannot communicate their feelings is currently unknown. It is also unknown whether and how their well-being and quality of life can be influenced. Well-being in this context refers to how children with CP are able to indicate they are enjoying life in their environments- ‘thriving or surviving’ which directly impacts upon their perceived quality of life. This research will observe children using the Innowalk, a robotic device as one context for them to indicate their well-being. The National Institute for Health and Care Excellence (2017) guidelines for the management of CP included recommendations to use validated measures to monitor their mental health and well-being, however available questionnaires are problematic for those who cannot communicate verbally or have a learning disability and experience epilepsy, fatigue or pain. Additionally, Mpundu-Kaambwa et al. (2018 10.1007/s11136-018-1837-0) did not find a valid and reliable measure of well-being for those with complex disabilities. However, a recent development by Oliver et al. (2020 10.1016/j.paed.2020.09.003), the Be-Well checklist for children with profound disabilities, has informed this study. Profound disabilities refer to those children with severe learning disabilities and complex needs. Other existing well-being measures will be reviewed in a co-productive way with children and their parents, to develop the domains for this new observational well-being scale for children with CP. This research will use the context of the Innowalk to observe well-being indicators in the children’s responses.

## A28 published 10.4081/audiores.2016.152

## A29 published 10.1186/s12998-021-00399-w

## A30 Service user evaluation of a large COVID-19 vaccination site in Wolverhampton

### ^1^Shehreen Gillani, ^2^Virinder Rai, ^2^Kam Ahmed, ^2^Erum Qureshi

#### ^1^St Peter's Collegiate Academy, Wolverhampton, United Kingdom. ^2^Unity West Primary Care Network, Wolverhampton, United Kingdom

Background: Novel coronavirus infection SARS-COV2 (Covid-19) was declared as Pandemic by WHO in 2020. UK rolled out mass vaccination program and has delivered over 139 million doses of Covid vaccines to people aged 12 years and over by February 2022. City of Wolverhampton delivered this program from 6 main sites and WV Active at Aldersley stadium was the largest site. We undertook a service user evaluation of this site to assess its effectiveness as a mass vaccination site. Methods: Engaging a group of people including staff members of the vaccination site, as well as service users, we developed 2 mixed questions proformas to use for this evaluation. One was used for people aged between 12 and 17 years (Group 1) and the other was used for people aged 18 years and above (Group 2). Each proforma was given to 100 consecutive users from group 1 and 150 consecutive users from group 2. User’s responses were recorded in MS Excel 2010 for analysis. Results: Group 1: Of 61 responses, 93% were aged between 12–15 years, 51% females, 70% White, 10% Asians and 3% Black. 59% were having their first vaccine dose. Rise in infection (56%) followed by travel (16%) were two top reasons for getting a jab. Users felt confident (9 ± 1(Median ± SD)), were happy with the information provided to them (9 ± 1.3), felt safe (98%) and rated their overall experience as positive (10 ± 0.9) with high recommendation rates (95%). Group 2: Of 92 responses, 89% were aged < 75 years, 50% females, 63% White, 16% Asians and 6% Black. 74% people were attending for their booster dose. People felt safe (89%) at site, rated location (46%), ease of booking (21%), walk in facility (20%) and parking availability (12%) as their priorities to choose a site with very high satisfaction (10 ± 0.6) and recommendation rates (99%). National Booking System (43%) was the highest route of booking into the service. Discussion: Among adults, ethnic minority attendance was in line with Wolverhampton demographics. However, in children ethnic minority attendance was significantly less in Black ethnicity. Although it is a very small sample size to draw any conclusions but it is in line with many other published evidence. All responses were significantly positive reflecting a well-organised and highly effective service delivered by well informed and supportive staff. Location of site and ease of parking were also marked as desirable characteristics of the site.

## A31 Outcome Measurement and Evaluation as a Routine practice in alcohol and other drug services in Belgium (OMER-BE)

### ^1^Charlotte Migchels, ^2^Amine Zerrouk, ^1^Frieda Matthys, ^3^Wim van den Brink, ^4^Lies Gremeaux, ^4^Kim Fernandez, ^2^Wouter Vanderplasschen, ^1^Cleo Crunelle

#### ^1^Vrije Universiteit Brussel (VUB), Universitair Ziekenhuis Brussel (UZ Brussel), Department of Psychiatry, Brussels, Belgium. ^2^Ghent University (UGent), Ghent, Belgium. ^3^Academic Medical Center, University of Amsterdam, Amsterdam, Netherlands. ^4^Sciensano, Brussels, Belgium

Introduction: In Belgium, we have a variety of specialised outpatient and residential Alcohol and Other Drugs (AOD) services, but little is known about their effectiveness and efficiency as research on outcomes of these services is limited. Patient-Reported Outcome Measures (PROMs) and Patient-Reported Experience Measures (PREMs) provide excellent tools and a framework to monitor progress and outcomes based on experiences of service users. Objectives: The OMER-BE project aims to: (1) Assess and compare patient characteristics at baseline in various treatment modalities; (2) Test and prepare the routine measurement and monitoring of PROMs and PREMs in AOD services in Belgium using a self-report tool; (3) Assess patient-reported experiences qualitatively in various treatment modalities for AOD patients in Belgium. The overall goal is to continuously assess and improve AOD services in Belgium. Methods: We will set up a naturalistic, longitudinal cohort study for which we will engage and follow up 250 AOD users as they present themselves in selected AOD services in five different treatment modalities (outpatient non-pharmacological treatment, outpatient substitution treatment, residential psychiatric treatment, therapeutic communities for addictions and mobile outreach teams). Sociodemographic, clinical, and intervention factors and PROMs will be assessed at baseline. PROMs and PREMs will be assessed at 45-, 90- and 180-days follow-up. The questionnaires that will be used during the baseline and follow-up assessments are based on the ICHOM Standard Set for Addictions (ICHOM SSA) (2020), a set of brief validated questionnaires to measure and monitor treatment outcomes routinely in AOD services. Following the 6-month follow-up we will perform a qualitative study in a subset of N = 25 participants (5 per treatment modality). These participants will be invited to take part in an in-depth interview with one of the researchers, where the following topics will be discussed: treatment history, recovery experiences, helping and hindering factors in recovery, and experiences with different treatment modalities.

## A32 Patient’s experience of their GP practice in the COVID-19 pandemic

### ^1^Paul Allanson, ^2^Paul Logan

#### ^1^University of Dundee, Dundee, United Kingdom. ^2^University of Edinburgh, Edinburgh, United Kingdom

The paper explores the impact of the COVID-19 pandemic on patients’ experiences of general practice in England using multicategory response data from the 2020 and 2021 GP Patient Surveys, where the former was conducted in the run up to the first UK national lockdown at the end of March 2020 and the latter a year later. It offers a novel analysis of changes in patients’ experience that is sensitive to changes in the distribution of patients across the full set of response categories, not just in the proportion meeting some binary quality threshold. The change in GP service quality nationally is measured as the difference in the chances that the overall experience of a randomly chosen patient in 2021 was better rather than worse than that of a similarly chosen patient in 2020. We similarly measure quality change at the individual practice level and break this down into a part attributable to the change in the national patient experience profile and a residual due to idiosyncratic practice-level profile changes, mirroring the distinction between structural and exchange components in the social mobility literature. Patients’ overall experience of their GP practice is shown to have improved with a 4.47 percentage point higher chance that a randomly chosen patient from anywhere in England in 2021 would have reported a better rather than worse overall experience of their GP practice than one similarly chosen in 2020. Practice-level changes exhibit reversion towards the median quality for England as a whole, likely reflecting the influence of transitory shocks to patient experience at patient and practice level, with the average change in patients’ rating of their own practice found to be slightly higher than the nationwide improvement due to the pattern of exchange mobility. Patients in 2021 were likely to rate their GP practice more highly if their last appointment was conducted face-to-face at their own practice rather than remotely over the phone or online. We conclude that patients’ more positive rating of their GP practice in 2021 was not a reaction to the prescribed switch towards the greater use of remote consultations, thereby contributing to the current debate on whether this change should be reversed once the pandemic is over. We conjecture instead that it was the result of a change in reporting behaviour stemming from a more supportive attitude towards the NHS during the pandemic.

## A33 published 10.1093/ndt/gfac170

## A34 Using machine learning models developed with English data to predict joint-replacement patient-reported outcomes in a cohort of patients from Cardiff and Vale UHB

### ^1^Aura Frizzati, ^1^Robert Palmer, ^1^Kathleen Withers

#### ^1^Cedar Health Technology Research Centre, Cardiff and Vale UHB, United Kingdom

Introduction: Aggregated patient reported outcome measure (PROM) records can be processed by supervised machine learning algorithms to create models to predict patients’ improvement after surgery. These predictions can support clinicians in management of patients’ surgery outcomes expectations. This project applied machine learning methods to predict improvement of post-operative PROM scores in a cohort of patients who underwent either hip replacement (HR) or knee replacement (KR) from Cardiff and Vale University Health Board (CAV UHB). Due to the small sample size of the CAV UHB dataset, the models were developed with English PROM data and then tested to predict improvement of CAV UHB post-operative PROM scores. Methods: Five classification algorithms were trained on 127,640 English PROM records (April 2016- March 2018) of HR and KR patients to predict post-surgical achievement of a minimal clinically important difference (MCID) for the EQ-5D’s visual analogue scale (EQ-VAS), the total Oxford Hip Score (OHS) and the total Oxford Knee Score (OKS). MCID is a binary variable and its post-surgical achievement was interpreted as patient improvement. The trained models were temporally validated on 63,269 English PROM records (April 2018- March 2019) and externally validated on 1,176 CAV UHB PROM records. Their accuracy was evaluated using the area under the receiver operating characteristic curve (AUROC) discrimination metric. Results: The classifier with the best discrimination performance at training was extreme gradient boosting. The most important predictor for EQ-VAS improvement was a patient’s pre-operative EQ-VAS. For total OHS and total OKS improvement the most important predictors were the pre-operative condition-specific PROM’s total score (total OHS or total OKS), the OHS or OKS limping dimension and the surgery revision status. The AUROC metric was higher when the models were tested on English data rather than on CAV UHB data. It was also higher for the models predicting post-operative EQ-VAS improvement in comparison to those predicting total OHS or total OKS improvement. Conclusions: Supervised learning classifiers were successfully developed using English records to predict improvement in post-operative PROM scores. Predicting improvement of EQ-VAS was easier (i.e. higher AUROC scores) than predicting total OHS or total OKS improvement. The predictive performance of the models when tested on CAV UHB data was worse than when they were tested on English data. These results suggest the need to develop predictive models directly on CAV UHB data to improve predictions in the CAV UHB cohort.

## A35 National evaluation of the ‘Adferiad’ (Recovery) Programme supporting the Welsh Long COVID Service

### ^1,2^Aura Frizzati, ^1,2^Robert Palmer, ^1^Megan Dale, ^1,2^Kathleen Withers, ^2^Sarah Puntoni

#### ^1^Cedar Health Technology Research Centre, Cardiff and Vale UHB, United Kingdom. Welsh Value in Health Centre, Cwm Taf Morgannwg UHB, United Kingdom

‘Long COVID’ refers to a wide range of signs and symptoms that persist or develop after acute COVID-19 illness caused by SARS-CoV-2 viral infection. It is not only associated with significant health and socio-economic harm for the affected individuals, but it also leads to an increase on NHS workload. In June 2021, the Welsh Minister for Health & Social Services announced the launch of the ‘Adferiad’ (Recovery) programme, funding the seven Welsh Local Health Boards (LHBs) to introduce a new suite of patient pathways combined with new/expanded primary and community rehabilitation services to support people with Long COVID. Welsh Government decided to review the programme every 6 months to monitor and assess the efficacy of the new services provided. Cedar Health Technology Research Centre and the Welsh Value in Health Centre (WViHC) supported the LHBs in responding to this request by facilitating data collection via a national survey, and by providing data analysis, reporting and summary at a national level. The data collected included patient-reported outcome measures (PROMs) and patient-reported experience measures (PREMs). A survey was administered via the internet to Long COVID service users from all LHBs and anonymised responses were collected by Cedar. The survey included 24 questions investigating service users’ demographics, their COVID-19 symptoms, the number of encounters they had with primary care, secondary care and rehabilitation services because of COVID-19, a measure of their generic quality of life (using the EQ-5D-5L PROM questionnaire) and their feedback on the Long COVID service (using a PREM questionnaire). Service users were divided into four groups, depending at which stage they were within the service (i.e. existing service users, new referrals, follow-up and discharged). Summary statistics were extracted from the quantitative data and statistical tests were carried out to identify any significant difference across the four groups in their PROM scores. PREM answers included free text data which was analysed via an inductive qualitative approach. This poster discusses the national evaluation report released by Cedar this year providing feedback on the service.

## A36 Development of an Inflammatory Bowel Disease (IBD) Patient-Reported Experience Measure (PREM): A patient-led consensus work and ‘think aloud’ study for a quality improvement programme

### ^1^Elena Sheldon

#### ^1^University of Sheffield, Sheffield, United Kingdom

Background: Patient-reported Experience Measures (PREMs) are key in improving healthcare quality, but no PREM exists for Inflammatory Bowel Disease (IBD). Objective: This study aimed to co-produce a PREM with IBD service users for IBD service evaluation and research. Design: Patient-led consensus work and a qualitative ‘think aloud’ interview study. Settings: The PREM was developed for an IBD service evaluation and quality improvement programme. Patients: IBD service users as experts and research participants. Main Outcome Measures: A pool of 75 items was drawn from published survey instruments covering interactions with services and aspects of living with IBD. In Stage 1, during two workshops, eight expert service users reduced candidate items through a ranked choice voting exercise and suggested further items. During Stage 2, eighteen previously uninvolved people with IBD assessed the face and content validity of the candidate items in ‘Think Aloud’ interviews. During two final workshops (Stage 3), the expert service users removed, modified and added items based on the interview findings to produce a final version of the PREM. Results: Stage 1 generated a 35-item working PREM mapped to the following four domains: Patient-Centred Care; Quality; Accessibility; Communication and Involvement. The PREM included a set of nine items created by the expert group which shifted the emphasis from ‘self-management’ to ‘living with IBD’. Stage 2 interviews showed that comprehension of the PREM was very good, although there were concerns about the wording, IBD-relevance and ambiguity of some items. During the final two workshops in Stage 3, the expert service users removed seven items, modified 15 items and added seven new ones based on the interview findings, resulting in a 38-item PREM. Limitations: The PREM’s reliability and validity remain to be established. Conclusions: This study demonstrates how extensive service user involvement can inform PREM development.

## A37 published 10.12688/wellcomeopenres.17518.1

## A38 Development of an implementation pilot to evaluate the feasibility and acceptability of the routine collection of patient-reported outcome measurements in a large NHS Cancer Centre

### ^1^Julie Malpass, ^2^Charlotte Moss, ^1^Garrard Knowles, ^1^Jenneh Bah, ^1^Gayla Hariram, ^1^Paula Treasure, ^1^Sheila Hassan, ^2^Mieke Van Hemelrijck, ^1^Ajay Aggarwal

#### ^1^Guy's and St Thomas' NHS Foundation Trust, London, United Kingdom. ^2^King's College London, London, United Kingdom

Introduction: Patient-reported outcomes (PROs) are an important component of clinical management providing insight into the health status of patients. PROs are generally measured using patient-reported outcome measurements (PROMs) which are validated self-administer questionnaire tools developed to assess various domains of quality of life. Whilst the benefits of utilising PROMs in the clinical setting are well-established in terms of shared decision making, their routine use is limited as healthcare services face increasing demand, financial deficits, and operational issues. This pilot was developed to identify barriers and facilitators to successful implementation of PROMs routinely within a large NHS Cancer Centre. Methods: The EPIC-26 PROM was selected for collection in a consecutive population of men with prostate cancer who had been referred for radiotherapy treatment. The men were approached in the clinical setting with a baseline PROM questionnaire prior to their end of treatment, and then were provided follow-up EPIC-26 PROMs for completion at 6 timepoints up to 2 years after end of treatment. Patients were given the option to complete the PROMs either on paper or electronically, with those opting for electronic emailed the follow-up questionnaires from a bespoke REDCap platform. All PROM data was entered onto the REDCap platform for ease of monitoring and analysis, and the project was conducted under local service evaluation. Results: In total, 20 prostate cancer patients were recruited and completed the baseline EPIC-26 PROM. Of the 20 men, 16 agreed to complete their follow-up PROMs electronically and 4 opted for pen and paper follow-up. As of 09/03/2022, 16 men had received their 6-week follow-up PROM and 11 had completed (68.75% compliance). Key barriers identified for successful implementation included availability of staffing resource, as this pilot relied on radiographers monitoring follow-ups whilst completing their usual clinical duties. Additionally, information technology was identified as a key barrier, as the REDCap platform formed an additional system for clinicians to access during their clinical consultations. Finally, the scoring system of the PROMs was also identified as a barrier, as there currently exists no consensus on threshold scores requiring further clinical management for patients with side effects as measured by the EPIC-26. Discussion: Overall, various barriers and facilitators have been identified and will be actioned as part of a wider implementation strategy at this Centre. A qualitative study, interviewing key stakeholders and patients involved in the pilot, is already on-going and will provide further critical data for this purpose.

## A39 The development and launch of a Canine Cruciate Registry using validated Client Reported Outcome Measures (CROMs)

### ^1,2^Mark Morton, ^2^Ashley Doorly, ^2^Amelia Poole, ^2^Chris Gush

#### ^1^ChesterGates Veterinary Specialists, Chester, United Kingdom. ^2^RCVS Knowledge, London, United Kingdom

Cranial cruciate ligament (CCL) disease is a common cause of lameness in dogs. In the UK the prevalence of diagnosis of CCL disease is reported at 0.56%. Two-thirds of these cases are managed surgically. There are numerous recognised surgical procedures, though there is a lack of high-quality evidence evaluating them in large populations of patients. The Canine Cruciate Registry (CCR) is an automated surgical registry that aims to collect anonymised data from patients across the United Kingdom. It is the first of its kind in veterinary medicine. It is open to all veterinary surgeons performing any technique. It is free to both veterinary surgeons and owners. It is funded by RCVS Knowledge (the independent charity partner of the Royal College of Veterinary Surgeons) avoiding any potential bias. Electronic consent is provided by both owners and surgeons. Following completion of a pre-operative baseline outcome measures, a surgical report form collects data about the procedure performed and owners are contacted regularly to complete follow up outcome measures. Outcomes are measured using LOAD (Liverpool Osteoarthritis in Dogs) and COI (Canine Orthopaedic Index), which are validated Client Reported Outcome Measures (CROMs) CROMs share similarities to proxy-Patient Reported Outcome Measures (PROMs). Complication reporting is available to both owners and surgeons. A friends and family test (FFT) equivalent is used; a Client Reported Experience Measure (CREM). Individual clinical audit is accessed via an online portal and anonymised data from the registry will be published in an annual report. Development of this registry has highlighted many similarities between registries and outcome assessment in human and veterinary patients. There is much we hope to learn from our human counterparts about engagement of both surgeons and owners, as well as data quality, analysis, and reporting. Likewise, as our project develops, we hope through ongoing collaboration, aspects of our experience may be mutually beneficial.

## A40 The Cancer Medicines Outcomes Programme (CMOP): Our Patient & Public Involvement Journey

### ^1^Emma Dunlop, ^2^Kelly Baillie, ^2^Julie Clarke, ^2^Jennifer Laskey, ^2^Jennifer McClintick, ^3^Fionagh Ross, ^4^Ally Boyle, ^4^Hugh Walker, ^1^Marion Bennie

#### ^1^University of Strathclyde, Glasgow, United Kingdom. ^2^NHS Greater Glasgow & Clyde, Glasgow, United Kingdom. ^3^NHS Lothian, Edinburgh, United Kingdom. ^4^Cancer Medicines Outcomes Programme, Glasgow, United Kingdom

Background: PROMs are firmly positioned within the Scottish Government’s cancer strategy. The Cancer Medicines Outcomes Programme (CMOP) is a Scottish Government funded national collaboration to report real-world outcomes of cancer treatments. One aim is to explore the feasibility of implementing PROMs into routine care to better inform treatment decisions; currently PROMs intelligence available is from clinical trials. In Phase 1 (2016–2020) we conducted early PROMs studies with clinicians, patients and carers around: what matters when discussing the impact cancer medicines have on quality of life; and the acceptability of technologies for collecting/using cancer medicines PROMs in routine care. We involved patients and carers as research participants, but recognised the need to involve them and the public in our decision making as our work streams progress in Phase 2. Our Approach: Protocols for recruiting Patient Representatives to the CMOP Programme Board, and establishing a Patient Network (where members contribute without the commitment of Board membership) were developed and outlined their roles and responsibilities, alongside a range of recruitment resources, adverts and social media posts, as well as support from a cancer charity, to aid engagement. In 2021 we recruited our first Patient Representatives and established our Patient Network. Our Patient Representatives are able to contribute at Programme Board meetings, and will be participating in the newly established Scottish Cancer PROMs Forum. This is an open collaborative space supported by Scottish Government and CMOP, for stakeholders (including patients, the public, the NHS and digital companies) to discuss and share learning across current PROMs research and work streams. This aims to minimise duplication of effort and patient population burden in the testing of PROMs digital tools, and contribute towards cancer and digital strategies. What Did We Learn?: Our efforts have been well supported by CMOP team and we received many notes of interest in the roles. The current Representatives show great commitment and enthusiasm to the programme, including PROMs specifically, and their contributions are welcomed by the Board. One team member undertook Patient & Public Involvement (PPI) training which was incredibly valuable. Patient involvement has reinforced CMOP’s priorities in keeping the patient/carer perspective at the heart of our thinking, influencing our direction of travel in all aspects of the programme, including PROMs. Next Steps: We are expanding our Patient Network and are recruiting Public Representatives to the Programme Board. We also plan to evaluate our PPI activities moving forward.

## A41 When is a patient a patient? Diagnosis validation in patient-centred research

### ^1^Sam Llewellyn, ^1^Catherine Bottomley

#### ^1^Vitaccess Ltd, Oxford, United Kingdom

A key part of the recruitment process for patient- and caregiver-reported studies is verifying eligibility of the prospective participant, referred to as diagnosis “validation”. This usually includes confirmation that the patient has a diagnosis of the disease of interest, but can also involve the validation of other participant inclusion criteria. Self- rather than clinician-led validation of diagnosis can be a necessary component of patient and caregiver research, for instance where participant recruitment is not based at a clinical site. To compare the advantages and disadvantages of self-confirmed diagnosis by patients against validation by a clinician, and to explore strategies to address known issues with the former. We use our experience in developing digital studies based on patient-reported outcome measures, in tandem with published literature, to provide a comprehensive assessment of patient-led validation of diagnosis. When compared with physician-led confirmation of diagnosis, self-validation is inexpensive, less time-consuming, and requires no external involvement. On the other hand, fraudulent patients may enrol to the study, and genuine patients may not be able to accurately report detailed eligibility criteria, such as taking a specific treatment or their disease stage. We propose strategies to address these concerns, namely: (1) monitoring data on an ongoing basis to filter out unusual response patterns that may indicate fraudulent participation; (2) including screening questions as a part of enrolment, to which only a true patient with the disease should be able to accurately respond; (3) recruiting via patient associations or support groups, where the pool of potential participants is highly likely to be genuine; (4) requesting potential participants to scan and/or upload a diagnosis letter or medication packaging as a part of enrolment; (5) using open methods of recruitment, such as general and social media advertising, with caution. For patient- and caregiver-reported studies where data from medical records are not required, speed and convenience are often favoured. As such, self-confirmation of diagnosis is frequently accepted by stakeholders. Several strategies can be put in place to address known issues with this method of diagnosis validation, thus improving studies by maximising the amount of meaningful data collected.

## A42 Using patient-reported data to estimate costs associated with melanoma in the UK: a digital registry

### ^1^Mishal Javed, *^2^Casey Quinn, ^2^Fatemeh Amini, ^2^Emily Boxell

#### ^1^Ministry of National Health Services Regulations and Coordination, Islamabad, Pakistan. ^2^Vitaccess Ltd, Oxford, United Kingdom

Background: Malignant melanoma is the fifth most prevalent cancer in the UK, and one of the leading cancers with regard to average years of life lost per death from disease. Despite this, there is a lack of data describing costs associated with melanoma for patients and the NHS. Aim(s): To estimate costs to the UK health system for the complete disease pathway in melanoma using real-world patient-reported data. Method(s): Data were principally from the Melanoma UK digital registry, an observational study collecting patient-reported real-world evidence on the impact of melanoma and its management through the My Melanoma study app (Vitaccess Ltd). Additional sources were used, such as UK NICE technology appraisals, for unit costs and in cases of limited data availability. A combination of top-down and bottom-up methods were applied to calculate costs for various domains of the disease pathway, including: drugs and administration, routine monitoring and management of disease, and management of treatment-related adverse events. Calculations were used to provide a snapshot of costs in the UK in 2019. Results: Taking into account several simplifying assumptions, the total costs associated with melanoma – calculated using data from 134 participants in the My Melanoma study – were estimated at £43,944 per patient, and £5.3 billion for the entire UK population. Of all domains, drug therapies were found to be the costliest, with approximately 79% of the total costs attributed to this domain alone. The costliest resource within each domain was: consultation with a medical oncologist (for routine disease management), treatment with ipilimumab (Yervoy®) (for monotherapy drugs), and treatment with a combination of nivolumab (Opdivo®) and ipilimumab (Yervoy®) (for combination therapy drugs). The cost for administration and dispensing per month of drug therapy was £232 for intravenous and £9 for oral drugs; the costs for a total of 1,390 adverse event episodes recorded in the registry were calculated at £529,105; and costs for routine disease management per month per patient were calculated at £440. Conclusions: We built a detailed, bottom-up picture of the per-patient costs to the UK health system of managing melanoma. To our knowledge, costing of the entire disease pathway in melanoma has not previously been attempted, nor costing at the level of the patient’s journey. These data and results should be considered a starting point: the entire patient journey is not fully captured, and much additional specificity can be developed with more data over time.

## A43 Mapping the evidence to identify outcome domains that are considered core to assessing the impact of adult specialist palliative care services in Wales

### ^1^Rhiannon Cordiner, ^1^Mala Mann, ^2^Anthony Byrne, ^2^Gladys Makuta, ^3^Rosemary Stewart

#### ^1^Specialist Unit for Review Evidence, Cardiff University, Cardiff, United Kingdom. ^2^Marie Curie Palliative Care Research Centre, Cardiff University, Cardiff, United Kingdom. ^3^Marie Curie Hospice, Cardiff and the Vale, Cardiff, United Kingdom

Introduction: Palliative care (PC) is a relatively new, expanding field in medicine that is looking for ways to develop in order to provide the best care for patients. The assessment of PC services for the development and enhancement of care delivery is crucial. The quality and effectiveness of PC services is becoming increasingly important to measure, as PC services have previously been assessed on mostly process related outcomes. A consensus driven approach has led to the formation of some quality assessment models (such as the PCOC^1^ in Australia and the OACC^2^ in the UK). However, it has been identified as a priority, that there is a need for a consensus driven approach for the assessment of PC services for Wales. Objectives: To identify the most important outcomes mentioned within the literature for evaluating the quality and effectiveness of PC services and to map these outcomes into common domain themes. The mapping will be reported in the format of a rapid review, which will then feed into an expert stakeholder consensus process. Method: Five databases were systematically searched. Journal searches were also carried out to supplement the papers identified. Adapted methodology from the Palliative Care Evidence Review Service (PaCERS)^3^ was used for this review. Results: Two hundred and fifty four articles were identified from the searches and nine of these met the pre-specified inclusion criteria. The most significant core outcome domains identified included: the structure and process of care, physical aspects of care and the psychological/psychiatric elements of care. Lesser mentioned domains included the social aspects and the ethical/legal elements of care. Conclusion: This review will feed into a future core outcome set consensus project and underpin the development of a future outcome measurement tool for the quality and effectiveness of PC service delivery in Wales.

**References**

1. Eagar, K, Watters, P, Currow, DC, et al. The Australian palliative care outcomes collaboration (PCOC)–measuring the quality and outcomes of palliative care on a routine basis. Aust Health Rev 2010; 34(2): 186–192.
2. Witt J, Murtagh FE, de Wolf-Linder S, Higginson IJ, Daveson BA. Introducing the Outcome Assessment and Complexity Collaborative (OACC) Suite of Measures-A Brief Introduction. Kings College London. 2014.
3. Mann, M., Woodward, A., Nelson, A. Byrne A. Palliative Care Evidence Review Service (PaCERS): a knowledge transfer partnership. Health Res Policy Sys. 2019;17(1):100. 10.1186/s12961-019-0504-4

## A44 published 10.1136/bmjopen-2021-057712

## A45 A data standardisation model for patient reported outcomes

### ^1^Gareth Griffiths

#### ^1^Digital Health and Care Wales, Cardiff, United Kingdom

Patient reported outcome measures (PROMs) are a fundamental component of a value-based healthcare approach. To achieve a truly data-driven decision making process underpinned by a value-based approach, PROMs information must be available to the right people, in the right place, at the right time. Although tools used to collect PROMs information define an agreed set of questions and possible answers, there is currently no mechanism for standardising the data captured across the range of organisations and applications that use these tools and, consequently, the data is often locked away in system and organisational silos. The standardisation model provides a basis for storing and communicating PROMs data records in a consistent way, regardless of the organisation or application. This serves as an enabler for interoperability and preservation of meaning across these boundaries, liberating the data for use in direct care, as well as in secondary uses population level analysis. The model is aligned to the principles of interoperability standards, such as HL7 FHIR and openEHR, to allow seamless and automated communication of PROMs data records. By applying these standards to a whole system architecture, the PROMs data record can be accessed wherever, and whenever, it is required. Fundamental to the model is the PROMs metadata set. Developed in collaboration with subject matter experts across NHS Wales, the PROMs metadata set is aligned to associated standards defined for clinical document repositories, meaning that completed PROMs forms can be indexed, enabling fast search and retrieval. The identifiers used anchor the patient’s PROMs record to their clinical record. They provide a mechanism for linking multiple completed PROMs forms together across the longitudinal health pathway and provide the capability for linkage to other data sources, such as event-based commissioning data sets, patient-level costing and clinical outcomes, to provide a holistic value-based picture. The standardisation model incorporates a codified, digitally consumable mapping for PROMs tools. These principles allow data gathered using any nationally validated PROMs tool to be stored and communicated in an efficient and standardised format. Each question is allocated a structured identifier which is unique across all nationally validated tools and questionnaires, meaning that they can easily be identified and categorised. This is particularly beneficial for the structuring of databases and messaging schemas. The possible answers to each question are also encoded for efficiency of storage and communication, whilst also providing simple mappings for use in national analytics, enabling powerful Wales-wide data comparability.

## A46 published http://dx.doi.org/10.1136/spcare-2021-MCRC.53

## A47 Radiomic Analysis of FDG PET-CT in Non-Small Cell Lung Cancer

### ^1^Khamael Albattat, ^1^Rhodri Smith, ^1^Nicholas Morley, ^1^Christopher Marshall

#### ^1^Cardiff University, Cardiff, United Kingdom

Lung cancer is responsible for a large proportion of cancer-related deaths, delayed detection is a significant factor for this high mortality rate. Radiomics is a high-throughput detection techniques of texture-features from medical images, has demonstrated excellent decision-making capacity for disease diagnosis and prognosis. Few studies have examined the radiomic signature of 18F-FDG PET/CT scan in early stage Non-Small Cell Lung Cancer (NSCLC). The present study was to retrospectively evaluate Radiomic Features (RFs) from18F-FDG PET/CT images, in a cohort of patients with early-stage NSCLC treated with radical radiotherapy to assess possible predictors and correlation with Overall Survival (OS) and clinical stage. A total of 105 patients were enrolled in the study each with histologically confirmed primary NSCLC. Tumour regions of interest (ROI) on PET images were semi-automatically segmented using region growing/thresholding approach. Texture features were extracted using an in-house MATLAB Programme. In total, 476 (2D&3D) RFs were extracted for each image. 167-3D features were chosen to complete the statistical analysis because the 3D analysis covers the full tumour volume and thus can better depict spatial heterogeneity. The correlation between the selected RFs and OS was examined with Spearman’s Rank correlation. A univariate cox regression analysis was performed to evaluate the selected RFs in predicting the primary endpoint OS. Subsequently, significant RFs with a p-value of < 0.05 from the univariate analysis was included in a multivariate cox regression to assess the Hazard ratio (HR) and corresponding 95% CI by considering nodal -ve patients as a control group. Multivariate cox regression analysis was fitted to the data based on clinical predictors only which are the clinical stage. Univariate analysis showed 17 RFs were significantly correlated with OS. Of these 9 features were chosen for cox regression i.e. those which had strong/very strong correlation with PET measures in spearman rank analysis (volume, GLCM-dissimilarity, GCLM-inv. Difference, GLRLM-percentage, GLSZM-lzhge, GLDZM-non-uniformity, NGTD-busyness, NGTD-complexity, NGLD-non-uniformity). From the cox regression analysis, one of the RFs were significantly predictive of OS, clinical stage did however provide a good predictor for the survival rate (nodal + ve group) (HR 3.078 P-value 0.009). An interesting finding in the analysis was the GLRLM-percentage had a HR of 6.3 which means the risk of death increased ~ 6 folds with this exploratory variable in the nodal + ve group. This result was not statistically significant, future work is obtaining more patient data to re-assess these results.

## A48 Evaluation of Patient and Public Involvement in the development of a patient reported outcome set in brain tumour trials (COBra Study)

### ^1^Elin Baddeley, ^1^Stephanie Sivell, ^1^Kathy Seddon, ^2^Helen Bulbeck, ^3^Ameeta Retzer, ^1^Annmarie Nelson, ^3^Melanie Calvert, ^4^Robin Grant, ^5^Richard Adams, ^6^Colin Watts, ^3^Olalekan Lee Aiyegbusi, ^3^Samantha Cruz, ^6^Pamela Kearns, ^7^Linda Dirven, ^1^Anthony Byrne

#### ^1^Marie Curie Palliative Care Research Centre, Cardiff University School of Medicine, Cardiff, United Kingdom. ^2^Brainstrust, United Kingdom. ^3^Centre for Patient Reported Outcomes Research, University of Birmingham, United Kingdom. ^4^The University of Edinburgh, Edinburgh, United Kingdom. ^5^Cardiff University, Cardiff, United Kingdom. ^6^University of Birmingham, Birmingham, United Kingdom. ^7^Leiden University Medical Center, Leiden, Netherlands

Introduction: Patient and public involvement (PPI) is increasing within health research internationally. Specifically within cancer research, PPI ensures treatment and care is in harmony with, and takes into account, the views, needs and preferences of those affected with cancer, and people with brain tumours are no exception. The COBra study is developing a core outcome set (COS) for use across research trials for adult glioma patients, while also identifying the outcomes that are patient reportable, of which a strong PPI influence is essential to ensuring the patient voice is incorporated. Aims: Describe the extent to which public contributors have influenced the COBra study, from conception, to date. Methods: Evaluate and explore public contributors’ involvement in the COBra study, retrospectively and prospectively, with the aid of a tool piloted by the study. The Public Involvement in Research Impact Planning and Tracking Pack, developed by researchers and public contributors at Marie Curie Palliative Care Research Centre and Wales Cancer Research Centre, uses the UK standards for Public Involvement, aimed to help plan, integrate, track, support and report public contributor involvement. Results: Two public contributors are co-investigators of the study and have been invaluable from conception. The UK Standards for Public Involvement have underpinned all involvement. Piloting the tool has allowed the consistent and comprehensive capturing of examples of the impact public contributors have made to the study. One important endpoint of the study is to identify patient reportable outcomes within the final COS, and our public contributors have been instrumental to working towards this endpoint. Specific examples of involvement and impact public contributors have made include 1. support of recruitment strategies of patients and caregivers to ensure their experiences are represented in the outcome list; 2. involvement in the interpretation of patient/caregiver interviews into outcomes; and 3. ensuring the outcomes and their definitions reflect the language used by the brain tumour community. Conclusion: Public contributors have been of utmost value to the study. Involvement has influenced and aided the extraction of outcomes from qualitative data, which is vital to ensuring patient voices are reflected in the outcome longlist, for the development of a Core Outcome Set for glioma research trials. Future involvement of public contributors will be invaluable to the continued activities of the study, including the consensus process, dissemination, ways to impact and uptake of the COS.

## A49 Developing Tools to Plan and Track Patient and Public Involvement (PPI) Impact in Research

### ^1^Alisha Newman, ^2^Alisha Newman, ^2^Julie Hepburn, ^2^Bob McAlister, ^2^Sarah Peddle, ^2^Kate Cleary, ^1^Annmarie Nelson

#### ^1^Marie Curie Palliative Care Research Centre, Cardiff, United Kingdom. ^2^Wales Cancer Research Centre, Cardiff, United Kingdom

Background: There is growing recognition that PPI positively shapes research and its relevance to the intended beneficiaries. The integration of PPI in research is becoming an increasing priority for funders and researchers alike. The UK Standards for Public Involvement in Research include impact. However, PPI can still appear to be a tokenistic exercise in some research projects. There is a recognised gap in practical tools to support the planned integration of meaningful PPI and to capture and demonstrate the difference PPI makes. Aim: To develop and test pragmatic tools to support researchers working with public contributors to: Plan and integrate public involvement in research, track public contributions and the difference they make to the research, support impact reporting against the UK Standards for Public Involvement. Methods: The Public Involvement in Research Impact Planning and Tracking Pack, ‘the pack’ was co-developed by public contributors and staff at the Marie Curie Palliative Care Research Centre and the Wales Cancer Research Centre. Regular project team meetings were held and working groups were convened to develop the resource and test its usability in practice. Results: The pack containing two tools, information about the UK standards for public involvement and signposting to other relevant PPI information was developed and tested with three Cardiff University led cancer focused studies, including one that seeks to develop a core outcome set (COS) for adult glioma patients, that takes into account patient reported outcomes (PROs). Preliminary feedback indicates that the tools are easy to understand and use. The planning tool is effective for mapping planned involvement activity and associated standards. Documenting involvement via the tracking tool is efficient when done routinely as contributions occur, and real time use was thought by researchers to make PPI feel genuinely integrated into practice instead of being bolted on. The tracking tool provides an impact focused framework that aids identification of specific public contributions that effect change. More information to help with interpretation of the standards in a study context was suggested to speed up the task of linking PPI activity to the standards. Some accessibility issues with the platform used for sharing/co-ownership of the tools were identified by public contributors. We are working towards refining the pack for a second test phase. When ready, the resource will be made widely available for use in research.

## A50 The development and initial impact of a cellulitis-specific Patient Reported Outcome Measure (CELLUPROM©) within the National Cellulitis Improvement Programme

### ^1^Marie Gabe-Walters, ^1^Melanie Thomas

#### ^1^Lymphoedema Wales, Swansea, United Kingdom

Introduction: Cellulitis is a bacterial skin infection with likely recurrence if not well managed. It can have devastating physical consequences, with a risk of sepsis if not appropriately treated. Anecdotal reports indicate that the impact of cellulitis extends beyond the acute symptoms to social and emotional morbidity; which may persist without intervention. The National Cellulitis Improvement Programme (NCIP) launched in Wales during 2020 under the auspices of Lymphoedema Wales. Initially, patients with a previous cellulitis-admission were reviewed by the NCIP, but more recently they are extending their remit into primary care. The NCIP provide an evidence-based intervention to reduce the risk of cellulitis recurrence, whilst improving patient and Health Care Professional knowledge. The NCIP has uniquely provided the opportunity to develop the first cellulitis-specific Patient Reported Outcome Measure (CELLUPROM©). Aims: To report on the provenance of CELLUPROM© and to examine its initial impact. Methods: CELLUPROM© was developed using an existing disease-specific PROM developed by Lymphoedema Wales. This PROM was updated to reflect existing literature and knowledge in cellulitis, along with expert review. As part of usual care, CELLUPROM© was completed before and after the NCIP. In late 2021, CELLUPROM© was adapted for use on a digital platform. Aggregate data are reported descriptively along with feedback. Results: Key stakeholders iteratively reviewed CELLUPROM© with key items added (e.g. fear of another episode of cellulitis), and others removed (e.g. shopping for clothes / shoes) or modified (e.g. finance / work). This gave rise to the 11-item CELLUPROM© with a free-text section for patients to report more widely on their impact of cellulitis. Each item is reported using an 11-point scale, where zero indicates no impact and 10 extreme impact. The biggest challenge reported by patients has been fear of a cellulitis recurrence. Positively, following the NCIP there was a significant decrease (M = 3.64 SD = 2.84) from baseline (fear M = 5.93 SD = 3.21), t(83) = 9.41, p < 0.001. Over 230 patients have completed CELLUPROM© on paper and a further 116 patients in the first three-months of digital collection. Conclusions: CELLUPROM© is an acceptable and feasible tool to help patients communicate their impact of cellulitis. The NCIP is optimising patient reported outcomes. As the NCIP expands into primary care, digital access using an automated platform grows increasingly important: enabling the timely collection of PROMs whilst supporting patients to complete at their convenience. Steps are to validate CELLUPROM© are already underway.

## A51 Evidence into practice and policy: PaCERS approach

### ^1^Mala Mann, ^1^Annmarie Nelson, ^1^Anthony Byrne

#### ^1^Cardiff University, Cardiff, United Kingdom

Introduction: The importance of linking evidence into practice and policy is accepted as a key pillar of a prudent approach to healthcare. However, rapid access to evidence to support policy and practice is a challenge globally. Rapid reviews (RRs) are increasingly employed as a research-synthesis tool to support timely evidence-informed decision making. There are various rapid review methods available with little or no guidance as to the format or content relating to conducting the review. Therefore, we established the Palliative Care Evidence Review Service (PaCERS)1 funded by Health and Care Research Wales through the Wales Cancer Research Centre, with the aim to support professionals and other decision makers working in palliative care delivering evidence in both a timely manner and usable format. Objectives: To describe development of the PaCERS methodology2, a service which is responsive to urgent, clinically driven, calls for research evidence to support service redesign opportunities or need for change to clinical care. Method: Our methodology was developed using systematic review methods to identify and appraise high quality evidence. In addition, a stakeholder workshop was held to refine our methodology and reporting processes and achieve consensus on how best PaCERS can serve the palliative care community. Results: To date we have produced eighteen evidence reviews. Findings will be presented from the point of engaging with requesters at the very start of the process to developing the review and the subsequent follow up to demonstrate impact. In addition, we will discuss the challenges involved in conducting rapid reviews and highlight methodology development unique to PaCERS. Conclusion: The principles of prudent healthcare should aim to underpin the advancement of services and close the gap between research and practice. Although, this service impact directly on palliative care clinicians and other decision makers, it effect patients/carers in receipt of palliative care. This approach could be adapted to suit partnerships between other healthcare disciplines and researchers.

## A52 Developing a patient-focused core outcome set for adult Palliative Care Services in Wales: A consensus-driven multi-stage project

### ^1^Silvia Goss, ^1^Stephanie Sivell, ^1^Elin Baddeley, ^2^Lowri Griffiths, ^3^Gladys Makuta, *^1^Anthony Byrne

#### ^1^Cardiff University, School of Medicine, Marie Curie Palliative Care Research Centre, Cardiff, United Kingdom. ^2^Marie Curie, Policy and Public Affairs Wales, Penarth, United Kingdom. ^3^Cardiff University, School of Medicine, Centre for Trials Research, Cardiff, United Kingdom

Introduction: Consistent assessment of palliative care service delivery is essential for driving improvements in care. Increasingly, there is recognition that such assessment needs to include measurements of the impact of services on patient- and family-focused outcomes, rather than just process-related outcomes. While the complexity and continuously changing nature of the needs of palliative care patients and their caregivers makes identifying appropriate outcomes difficult, there have been successful local initiatives to establish standardised core outcome sets, such as the PCOC in Australia and the OACC in England. The End-of-Life Care Board (EoLB) in Wales now seeks a similar identification of key outcomes for adult palliative care services (PCS), establishing consensus on key domains of importance and determining if existing approaches are appropriate to capture these within the health and social care economy specific to Wales. Objectives: To establish a consensus on a core outcome set for adult palliative care services that best reflects patient- and family-focused assessments of quality and effectiveness of care in Wales. Method: This project is a multi-stage study. Stage I: A rapid review of existing literature to identify key outcomes already used in the UK and internationally and to map those into key domains of service quality and effectiveness in palliative care. Stage II: An expert group workshop to consider the rapid review evidence, identify any gaps and agree on a longlist of outcomes. Stage III: An online survey of this longlist with outcomes to be ranked by importance by a wider variety of stakeholders. Stage IV: An expert meeting to reach consensus on the final core outcome set for adult PCS in Wales and to discuss whether existing assessment approaches (e.g. OACC, PCOC) capture these outcomes adequately. Results: This project is ongoing, with Stages I and II recently completed. A longlist of 61 outcomes across 10 domains was identified in Stage I and is currently being refined based on the expert feedback received in Stage II. The Wales-wide stakeholder online survey to obtain a shortlist of outcomes through ranking will be held in April (Stage III). Following this, the key outcome measurements for Wales will be agreed in May (Stage IV). At the conference, we will thus present the final core outcome set proposed for Wales. Conclusion: This consensus-driven project will directly underpin and inform the EoLB’s judgement on a future core outcome measurement set for adult PCS, refined to best reflect Wales’ needs.

## A53 Experiences of developing a treatment-specific Patient Reported Outcome Measure for the impact of glucocorticoid therapy using an international online survey

### ^1,2^Susan Bridgewater, ^1,2^Mwidimi Ndosi, ^1,2^Joanna C. Robson

#### ^1^University of the West of England, Bristol, United Kingdom. ^2^University Hospitals Bristol and Weston NHS Foundation Trust, Bristol, United Kingdom

Background: Glucocorticoids (GCs) are a key treatment for inflammatory rheumatic diseases, but they cause a range of adverse effects of concern to patients and clinicians. Objectives: This project is to develop a Patient-Reported Outcome Measure (PROM) for glucocorticoids (the Steroid PRO). We report here our experiences of patient recruitment to an online observational survey. Methods: Underpinning qualitative interviews with 60 patients from the UK, USA and Australia have previously informed a long-list of 40 candidate questionnaire items for the Steroid PRO. These items have undergone piloting with patient partners, cognitive interviews and linguistic evaluation. This study is a large-scale online survey to test the draft Steroid PRO and determine the final scale structure and measurement properties. We collaborated with patient groups in the UK, USA and Australia who advertised the survey link with their members via email contact lists and social media platforms. The survey was anonymous. Adults currently taking glucocorticoids for rheumatic disease were eligible to participate. Patients gave implied consent by completing the survey. The survey included the draft Steroid PRO and generic EQ5D-5L questionnaires, questions about rheumatic disease (diagnosis, GC dose) and demographics (age, gender, country of residence, ethnicity, educational level). Participants had the option to receive a follow-up survey link 3–5 days after baseline for a repeat Steroid PRO questionnaire and change of state questions. Results: The large-scale online survey was shared with over 20 patient groups to disseminate via email, Twitter, Facebook and Instagram. Observations from the research team suggest that postings on private Facebook groups were the most effective social media recruitment method. Screening questions were used to ensure data quality; 25 participants were excluded due to not having glucocorticoids in the last week. The survey received 1,748 initial page views, 946 with complete responses (all questionnaire items and demographics). Of these, 833 (88.1%) participants were female; country UK n = 743, USA n = 139, Australia and New Zealand n = 64. Diagnoses: Inflammatory Arthritis n = 197; Connective Tissue Disease/Vasculitis n = 402; Giant Cell Arteritis/Polymyalgia Rheumatica n = 346. The follow-up survey was completed by 481 (51.1%) participants. Conclusion: This international online survey progresses the development and validation of the Steroid PRO. Next steps include validation with Rasch models and factor analysis to determine scale structure and measurement properties. Following Covid-19, online patient recruitment is an increasingly used research tool and can be very effective. Methodological challenges, e.g. selection bias and data quality, need to be considered in future studies.

## A54 Recovery goal menu development to be utilised with Goal Attainment Scaling for adult survivors of critical illness

### ^1,2^Chloe Apps, ^1^Kate Brooks, ^2^Ella Terblanche, ^1,2^Nicholas Hart, ^1^Joel Meyer, ^2^Louise Rose

#### ^1^Guy's and St Thomas' NHS Foundation NHS Trust, London, United Kingdom. ^2^King's College London, London, United Kingdom

Introduction: Recovery goal setting is a central feature of our digital ICU recovery pathway that we designed to support ICU survivors’ transition from hospital to home. Recovery goal setting utilises Goal Attainment Scaling (GAS). GAS is an individualised patient-reported outcome measure (PROM) comprising goal setting with achievement self-reported by patients. Goal setting with GAS can be time-consuming to complete, therefore condition-specific goal menus have been created. To date, no goal menu for adult ICU survivors has been developed. Objective: To develop an expert and end-user informed recovery goal menu to be embedded in our digital ICU recovery pathway for use by ICU survivors. Methods: We are developing the goal menu in two stages. Stage 1 (complete) consisted of three consultation meetings with ICU survivors, relatives, and experts in ICU recovery to gain consensus on the theoretical framework underpinning the menu, menu item generation, and refinement of wording. In Stage 2 we will establish content validity through cognitive interviews with our key stakeholder groups to assess item: i) relevance, ii) comprehensiveness, and iii) comprehensibility following the COnsensus-based Standards for the selection of health Measurement INstruments (COSMIN) recommendations. Results: We report stage 1 results. At meeting 1 attended by 7 ICU recovery experts, we gained consensus to use the Functional Independence Measure and Functional Assessment Measure (FIM FAM) and the Canadian Model of Occupational Performance and Engagement (CMOP-E) as menu theoretical underpinnings. At meeting 2 attended by 2 end users, agreement was reached on 4 domains (Self-care, Productivity, Leisure, Person). Menu item generation discussion resulted in the following: Self-care domain: 33 items, Productivity domain: 10 items, Leisure domain: 13 items and Person domain 17 items. At meeting 3 attended by a further 2 end users, 4 additional items were proposed for the Self-care domain, 3 items for Productivity domain, 12 items for Leisure domain, and 3 items for the Person domain. Examples of items suggested include stoma care, going to the gym and acceptance of scars. One item was modified in the Self-care domain. No items were considered redundant. On Stage 1 completion the menu now comprises Self-care domain: 37 items, Productivity domain: 13 items, Leisure domain: 25 items, and Person domain 20 items. Conclusion: We developed an expert and end-user informed recovery goal menu specific to the needs of ICU survivors’ transition from hospital to home. Further work will confirm content validity and feasibility in clinical practice.

## A55 An analysis of baseline electronic patient-reported outcome measures (ePROMs) of patients with lung cancer treated at the Christie NHS Foundation Trust

### ^1^Cathryn Crockett, ^2^Danya Abdulwahid, ^2^Neil Bayman, ^2,3^Fiona Blackhall, ^2,3^Raffaele Califano, ^2^Clara Chan, ^2^Joanna Coote, ^2^Laura Cove-Smith, ^2^Fabio Gomes, ^2^Margaret Harris, ^2^Sarah Hughes, ^2^Colin Lindsay, ^2^Laura Pemberton, ^2^Shereen Rafee, ^2^Hamid Sheikh, ^2^Yvonne Summers, ^2^Paul Taylor, ^2^David Woolf, ^2,3^Corinne Faivre-Finn

#### ^1^Northern Ireland Cancer Centre, Belfast, United Kingdom. ^2^The Christie NHS Foundation Trust, Manchester, United Kingdom. ^3^The University of Manchester, Manchester, United Kingdom

Background: The online ePROM platform ‘MyChristie-MyHealth,’ was introduced at the Christie NHS Foundation Trust in January 2019. It currently enables routine remote completion of symptom and quality of life (QoL) questionnaires by patients from multiple disease groups, including lung cancer. Responses are used to offer patients symptom advice and also to better inform subsequent hospital consultations. Methods: A database containing ePROM responses and demographic/clinical information of lung cancer patients between January 2019 and December 2020 was created. Here we report an analysis pertaining to ePROMs completed at the time of the first consultation, prior to commencing any treatment (‘baseline’). Results: 1480 patients with lung cancer completed ePROMs during their treatment pathway. Of those, 378 completed a baseline ePROM. The median age was 67 years (range 27–88 years), 51% were male respondents, the majority of patients had good ECOG performance status (PS [0–1, 57.4%]), no or few co-morbidities (Adult Co-morbidity Evaulation-27 score [ACE-27] 0–1, 49.7%) and a smoking history (63% current or ex-smokers). Most had non-small cell lung cancer (71.1%) and 41.5% had stage IV cancer. The mean symptom scores at baseline for this patient group were highest for pain, dyspnoea, cough, fatigue, and anorexia. This is a predictable result given these symptoms are most likely attributable to lung cancer and/or an associated comorbidity. Also as expected, the scores for nausea, vomiting, diarrhoea, constipation, and paraesthesia, were low prior to commencement of treatment. There were no significant differences in baseline symptom and QoL scores when compared on the basis of patients’ age (< 70 vs. ≥ 70). However, in patients with higher ACE-27 scores (2–3 vs. 0–1), dyspnoea (p = 0.035), haemoptysis (p = 0.023), nausea (p = 0.041), mobility (p = 0.004) and self-care (p = 0.0420) were all significantly worse. An unexpected finding was that baseline cough (p = 0.006) and mobility (p = 0.006) were significantly worse for patients with better ECOG PS (0–1 vs. 2–3). Conclusion: Our analysis has shown that the patients who completed ePROMs were younger, fitter and healthier than the average lung cancer patient we typically see in our clinics. It has also highlighted that the Christie lung cancer-specific ePROM questionnaires appear to demonstrate adequate validity, given their findings regarding baseline symptoms and QoL scores are for the most part as expected in this patient population.

## A56 published 10.1308/rcsann.2022.0041

## A57 Measuring dental anxiety before dental treatment in paediatric patients using the Modified Child Dental Anxiety Scale (MCDASf)

### ^1^Marija Borisovaite-Petruliene, ^1^Richard Balmer, ^1^Theresa Munyombwe, ^1^Collette Gardener

#### ^1^University of Leeds, Leeds, United Kingdom

AIM: To evaluate and compare dental anxiety in paediatric patients having dental treatment under general anaesthesia (GA) or with inhalation sedation (IHS) using psychological measure (MCDASf) before dental treatment. METHODS: The population of the study was 80 paediatric patients aged 6–15 years who were treated under GA or IHS. Faces version of the Modified Child Dental Anxiety Scale (MCDASf) was used to measure self-reported dental anxiety before dental treatment under GA or with IHS. Data was analysed using IBM SPSS Statistics 26.0 programme. RESULTS: Of 80 participants included in the study, 48 (60.0%) were female and 32 (40.0%) were male, average age of 8.44 years SD =  ± 1.97. A total of 57 patients (71.3%) had their treatment under GA while 23 patients (28.7%) were treated with the help of IHS. The baseline MCDASf scores in GA group had a mean score of 21 SD =  ± 6.02. The baseline MCDASf scores in IHS group had a mean score of 24 DS =  ± 5.39. Anxiety score was higher in IHS patients (mean difference 2.79 (95% CI, -0.13 to 5.73) p = 0.06); however, it was not statistically significant. The most common components to rate highly in causing anxiety were ‘having an injection in the gum’ [n = 53, 66.3%] and ‘having a tooth taken out’ [n = 45, 56.3%]. These were given scores of more than 3 indicating ‘a lot of worry’ by 53 and 45 of the 80 patients completing the questionnaire before their treatment. CONCLUSIONS: Although MCDASf questionnaire demonstrated that children were more anxious before their treatment session with inhalation sedation when compared to general anaesthesia, it was not significant. According to the results of MCDASf questionnaire, most of the patients were highly anxious of having local anaesthesia and tooth extraction. LIMITATIONS: The current results are preliminary findings and sample sizes in both groups differ at this early stage of the study.

## A58 Measuring experiences of making decisions about research on behalf of others: development of the self-reported Combined Scale for Proxy Informed Consent Decisions (CONCORD)

### ^1^Victoria Shepherd, ^1^Kerry Hood, ^1^Fiona Wood, ^2^Katie Gillies

#### ^2^Cardiff University, Cardiff, United Kingdom. ^3^University of Aberdeen, Aberdeen, United Kingdom

Background: The quality of care provided for people with cognitive impairment is impacted by the research inequalities that these groups experience. People with cognitive impairment may be unable to provide informed consent and are frequently excluded from research, leading to ‘evidence-biased’ care. This may include people living with dementia, learning disabilities, are critically ill, or other groups unable to participate in decisions about their care. Interventions are needed to ensure these under-served groups have better opportunities to participate in research and improve future care. People with impaired capacity to consent require the involvement of alternative ‘proxy’ decision-makers, usually a family member. It can be challenging experience for family members, with some experiencing an emotional and decisional burden. To date, the lack of validated instruments to measure proxies’ experiences of consent decisions limits our ability to evaluate interventions to support families making proxy consent decisions. This presentation outlines the development of the Combined Scale for Proxy Informed Consent Decisions (CONCORD). Methods: A four-stage process was used to develop and refine items for a new measure of proxy decision-making: 1) content generation and review of existing measures; 2) assessment of content coverage by existing measures and identification of (in)sufficiency; 3) construction of a novel scale; 4) cognitive testing to explore comprehension of the scale and test its content adequacy through interviews with family members of people with impaired capacity. Results: Core outcomes established through a recent scoping review and consensus study were reviewed to identify items for inclusion in the measurement scale. A range of outcome measurement instruments associated with healthcare decision-making and informed consent decisions were identified and mapped against the key constructs such as values clarity, understanding, preparedness, and decisional regret and satisfaction to assess content coverage. Insufficient coverage indicated that a novel measure was needed. An initial version of a combined measure (the CONCORD scale) was drafted, covering proxies’ preparation for decision-making, decision-making process, and decision outcome. It was tested with eleven family members of people with impaired capacity to assess comprehension, acceptability, feasibility, and content adequacy, leading to the creation of a revised version. Conclusions: CONCORD provides a measure of families’ experience of decision-making on behalf of an adult who lacks capacity, enabling the evaluation of interventions to support proxy decisions and improve inclusion of this under-served group in research. Initial evaluation indicates content adequacy, acceptability, and feasibility. Further work to concurrently validate the scale is underway.

## A59 Welcome to the FORUM: A new patient and clinician reported outcome measure for forensic mental health services

### ^1^Howard Ryland, ^1^Jonathan Cook, ^1^Raymond Fitzpatrick, ^1^Seena Fazel

#### ^1^University of Oxford, Oxford, United Kingdom

Forensic mental health services provide care to people with severe mental illness who also pose a risk to others, in secure psychiatric hospitals and specialised community teams. Measuring outcomes of forensic mental health services is important to safeguard patients and the public, monitor progress and develop treatment plans. Little is known about which outcomes are most important and existing measures have had limited patient input into their design, demonstrate variable psychometric properties, and are often not well liked by clinicians. A project to develop a new outcome measure was co-designed with patients, carers and the public. A dedicated patient and public advisory group was established to guide the work throughout its lifecycle and contributed to project methodology, data interpretation and dissemination of results. Patients, carers and professionals from forensic mental health services were interviewed and took part in focus groups to identify which outcomes were important to them. Forty-two outcomes were identified in the six domains of ‘about me, my quality of life, my health, my safety and risk, my life skills and my pathway’. These outcomes were then prioritised by asking patients, carers and professionals to rate their importance through a Delphi process. Eight of the top fifteen outcomes were shared between patients/carers and professionals. A new instrument for measuring outcomes in forensic mental health services was then developed, called the FORensic oUtcome Measure (FORUM), with complementary patient (FORUM-P) and clinician (FORUM-C) reported questionnaires. Patients and their clinical teams at a regional forensic psychiatric service then completed the FORUM. Patients and clinicians also provided feedback on the questionnaires. Sixty-two patients participated with a mean age of 41.0 years (standard deviation 11.3) and 35 clinicians completed the FORUM-C. Cronbach’s alpha for the FORUM-P was 0.87 (95% confidence interval (CI) 0.80–0.93) and the FORUM-C was 0.93 (95% CI 0.91–0.96). The weighted kappa for test–retest reliability for the FORUM-P was 0.44 (95% CI 0.24–0.63) and for the FORUM-C was 0.78 (95% CI 0.73–0.85). For interrater reliability of the FORUM-C the Spearman correlation coefficient was 0.47 (95% CI 0.18–0.69). The FORUM-P received an average rating of 4.0 out of 5 for comprehensiveness, 4.6 for ease of use and 3.9 for relevance, while the FORUM-C received 4.1 for comprehensiveness, 4.5 for ease of use and 4.3 for relevance. These results indicate that the FORUM is a promising new instrument to measure outcomes in forensic mental health services.

## Data Availability

Data sharing not applicable to this article as no datasets were generated or analysed during the current study.

